# Integrated Bioinformatic Analyses Constructed a Novel Immune Escape‐Related Signature and Classifier to Predict Tuberculosis

**DOI:** 10.1111/jcmm.70562

**Published:** 2025-04-27

**Authors:** Zhenpeng Li, Yixin Xu, Huizi Zhou, Wentao Wang, Haien Cheng, Meng Li, Aili Chen, Chao Zhao

**Affiliations:** ^1^ School of Medical Laboratory Shandong Second Medical University Weifang Shandong China; ^2^ Engineering Research Institute of Precision Medicine Innovation and Transformation of Infections Diseases Shandong Second Medical University Weifang Shandong China; ^3^ School of Clinical Medicine Shandong Second Medical University Weifang Shandong China; ^4^ Office of Academic Affairs Shandong Second Medical University Weifang Shandong China

**Keywords:** biomarker, immune escape, machine learning, subgroups, tuberculosis

## Abstract

Despite its high preventability and curability, tuberculosis (TB) remains a leading cause of morbidity and mortality worldwide. One factor that contributes to the susceptibility and progression of various diseases is immune escape. Therefore, the primary aim of our study was to explore the involvement of immune escape‐related genes in the pathogenesis of TB. Two TB datasets retrieved from the gene expression omnibus database were used to identify differentially expressed genes (DEGs). Machine learning was used to identify the hub immune escape‐related genes (HIERGs). Weighted gene co‐expression network analysis supported and further validated these findings. Subsequently, we scrutinised two distinct subgroups that were determined through the identification of hub immune escape‐related genes, and evaluated the distinct function of the subgroups. Our study identified a total of 11 genes related to immune escape in TB. Additionally, six HIERGs were identified through the least absolute shrinkage and selection operator (LASSO) and support vector machine‐recursive feature elimination (SVM‐RFE) algorithms. Diagnostic models constructed using HIERGs exhibited high accuracy. Two immune escape‐related subclusters were identified in TB samples, which delineated differences in immune infiltration cells with the distinct TB subgroups. The heightened expression of six HIERGs serves as a significant risk factor for TB. The six HIERGs also contribute towards the development of TB‐related diseases. Our findings demonstrate a significant enrichment of immune escape‐related gene expression in individuals with TB, suggesting a close relationship between immune escape activity and immune cell abundance. These results underscore the putative role of immune escape in the advancement of TB by disrupting or perturbing the immune response.

## 1 Introduction

Tuberculosis (TB), which is caused by *Mycobacterium tuberculosis* (Mtb) infection, continues to be an enduring worldwide health concern. It is estimated that approximately one‐quarter of the world's population is infected with Mtb [1]; Mtb remains latent in most individuals because of immune suppression [2]. In a small proportion of individuals, however, Mtb overcomes immune defences and causes progressive disease marked by bacterial replication and inflammation, a potentially lethal outcome if left untreated. Prior to the novel coronavirus pandemic, active TB held the dubious distinction of being the leading infectious cause of death, and the disease is still a major global public health menace [3]. Early and accurate diagnosis of TB is critical in enhancing patient care and improving patient outcomes.

By eluding the host immune response, Mtb is capable of establishing persistent infections. Mtb utilises various strategies to subvert the host's immune system to favour persistent infection and disease progression via ubiquitination [4, 5], PE_PGRS [6, 7], TNF receptor‐associated factor 6 [8], the complex cell‐wall structure [9, 10], Mtb‐infected foamy macrophages [11, 12, 13] and others. Although numerous immune escape mechanisms have been identified to date [14, 15], these likely represent just the proverbial tip of the iceberg. Identifying  ssential pathogen virulence regulators and researching the underlying immune response are both crucial to developing more effective vaccines and therapeutic targets. This includes identifying immune escape‐related genes responsible for poor TB prognosis, which is an essential strategy for evaluating and enhancing the effectiveness of immunotherapy.

Although immune escape plays a key role in TB, few studies have revealed and established immune escape models to improve the accuracy of diagnosis and prognosis. Therefore, this study aims to identify and construct a novel immune escape‐related signature, and this signature is expected to provide a theoretical basis for further understanding the mechanism of immune escape in the process of TB and identify potential biomarkers, thereby deepening our understanding of the molecular mechanisms of TB at the systems biology level.

## 2 Material and Methods

### 2.1 Study Design

**FIGURE 1:**
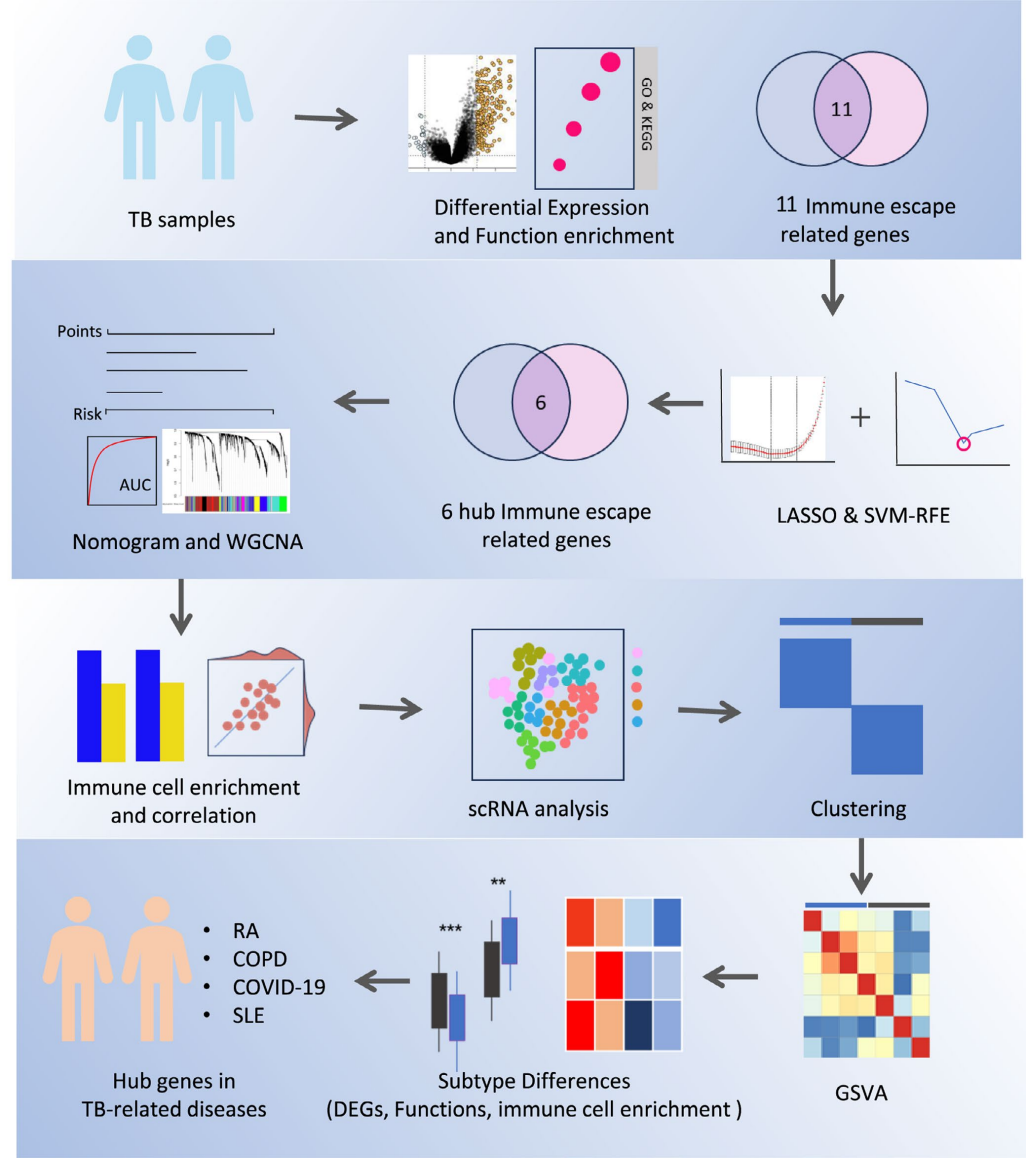
Workflow of this study. Differential gene expression and functional enrichment analysis; machine learning for hub gene selection and diagnostic model development; analysis of the relationship between hub genes and immune cells, and validated through single‐cell RNA analysis; TB samples clustered and gene set variation analysis (GSVA), identification of DEGs, functional analysis and analysis of immune cell infiltration; hub genes analysed in TB‐related diseases.

The primary workflow of this study was given in Figure 1.

### 2.2 Collection of Datasets

The Gene Expression Omnibus (GEO, www.ncbi.nlm.nih.gov/geo) website database was used to retrieve the GEO datasets (GSE83456, GSE62525, GSE93272, GSE76925, GSE166253 and GSE50772); The samples included patients who (1) were > 15 years old, (2) were diagnosed according to their respective criteria, (3) had a single disease consistent with the research objectives and (4) had been validated in other articles. In addition, the number of samples for each type in every gene expression profiling dataset must be > 5, and the samples must consist of RNA expressed at the whole human genome level. The detailed information about the GEO datasets was provided in Table S1. Furthermore, we obtained a total of 182 immune escape genes from a prior investigation (Table S2) [16, 17].

### 2.3 Analysis of Differentially Expressed Gene

The ‘limma’ package was used to identify DEGs. Genes that demonstrated a log2|fold change| > 0.585 and a *p*‐value < 0.05 were deemed to be statistically significant. We obtained the expression profile datasets, performed multivariate linear regression using the lmFit function, and further used the eBayes function to modulate the standard errors to a common value through the empirical Bayesian method.

### 2.4 Functional and Pathway Enrichment Analyses

We conducted Kyoto Encyclopedia of Genes and Genomes (KEGG) enrichment and Gene Ontology (GO) analyses utilising the ‘clusterProfiler’ R package [18]. We utilised GSVA to elucidate functional variations between subclusters that were identified in the earlier cluster analysis. For GSVA analysis, we downloaded files ‘h.all.v7.5.1.symbols’, ‘c2.cp.kegg.v7.5.1.symbols’ and ‘c2.cp.reactome.v7.5.1.symbols’ from the MSigDB online database.

### 2.5 Machine Learning

Based on the expression profiles of IERGs, we developed the LASSO model [19]. The smallest lambda value serves as a benchmark for identifying the optimal parameters. We utilised regression coefficients derived from LASSO analysis to assign weights to the expression levels of the chosen genes. The model produced with these ideal lambda values was scrutinised, and we computed the regression coefficients for every gene. Subsequently, any gene with a coefficient of zero was excluded, and the remaining coefficients were employed to construct our diagnostic model. The analysis of LASSO regression was performed using the R package ‘glmnet’. In addition, SVM, a supervised machine learning technique, integrates the principles of support vector machine‐recursive feature elimination (SVM‐RFE). This backward search method diminishes spatial dimensions by discarding superfluous features, facilitating the discovery of genes with the highest discriminatory potential and the categorisation of pertinent attributes. The SVM‐RFE algorithm was executed utilising the e1071 package within the R programming environment [20]. Intersection genes were considered hub genes.

### 2.6 Immune Cells Assessment

CIBERSORT algorithm was utilised to calculate the proportionate amounts of 22 immune cell subsets based on gene expression [21]. Subsequently, we filtered out the immune cells with significant infiltration differences between TB and HC, and analysed their correlation with characteristic genes.

### 2.7 Weighted Gene Co‐Expression Network Analysis (WGCNA)

WGCNA on DEGs through the utilisation of the R package ‘WGCNA’ [22]. To guarantee that the constructed co‐expression network approached a scale‐free distribution, we set 3 as the soft power. Through this process, we derived 15 modules and assessed their correlation with the cluster.

### 2.8 Single‐Cell Data Preprocessing

Single‐cell RNA‐seq libraries of modes were constructed following the 10× Genomics protocol that was previously described [23, 24]. We employed the FindCluster package for all cell cluster analysis, with a resolution set to 0.5. In Table S3, we have listed the marker genes for each cluster [25].

### 2.9 Subclusters Analysis With Six HIERGs

For the 92 TB samples used in this study, we performed an unsupervised hierarchical clustering analysis to identify the mRNA expression of six HIERGs, using the ‘ConsensusClusterPlus’ R package [26] as the analytical tool.

### 2.10 Quantitative Real‐Time PCR (qRT‐PCR)

A total of 16 plasma samples (eight TB and eight HC) were collected from the affiliated Hospital of Shandong Second Medical University. Informed consent was obtained from all patients prior to beginning the study. Total RNA was extracted using TRIzol (Invitrogen) according to established protocols. Complementary DNA (cDNA) was obtained using the HiScript III RT SuperMix for qPCR (+gDNA wiper) (Vazyme, NanJing, China). Quantitative real‐time PCR (qRT‐PCR) was conducted with the ChamQ Universal SYBR qPCR Master Mix (Vazyme), following the manufacturer's instructions and recommended cycling conditions. The relative expression levels of target transcripts were calculated using the 2‐ΔΔCt method, which normalises the data to the reference gene GAPDH. The primer sequences employed in this study can be found in Table S5.

### 2.11 Statistical Analysis

The statistical analyses were completed utilising R (4.2.2) software. Adobe Illustrator (CC 2020) was employed to compile the figure panels. The t‐test was used to analyse boxplots. For correlation analysis, the Spearman's coefficient was used. All statistical tests were two‐sided, and a *p* value or adjusted *p*‐value < 0.05 was considered to be statistically significant.

## 3 Results

### 3.1 Differentially Expressed Genes Identified

**FIGURE 2:**
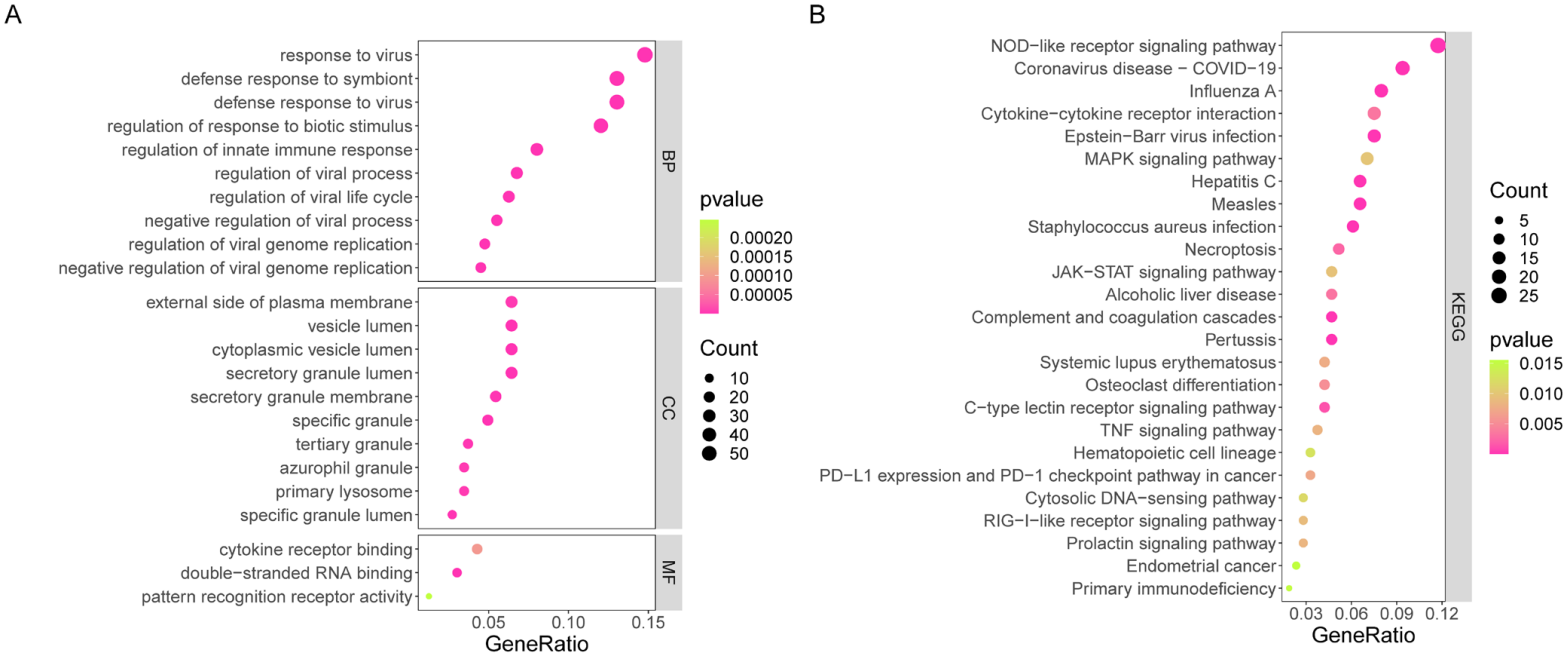
Functional enrichment of DEGs. (A) GO analysis (B) and KEGG analysis of the DEGs. DEGs, differentially expressed genes; GO, Gene Ontology; KEGG, Kyoto Encyclopedia of Genes and Genomes.

A total of 628 DEGs (349 upregulated and 279 downregulated) were identified between patients with TB and HC in the GSE83456 dataset. To explore the function of these DEGs in TB development, the 628 DEGs underwent GO and KEGG analysis. GO enrichment analysis showed that the DEGs were largely enriched in virus infection and immune‐related biological processes (Figure 2A). The DEGs were mainly located on the external side of the plasma membrane, and their molecular functions were cytokine receptor binding (Figure 2A). KEGG analysis also revealed that virus infection and immune‐related signalling pathways were the most enriched (Figure 2B). These findings suggested that these DEGs play a crucial role in virus infection, immune response and disease regulation.

### 3.2 Identification of a six Immune Escape‐Related Signature for TB

**FIGURE 3:**
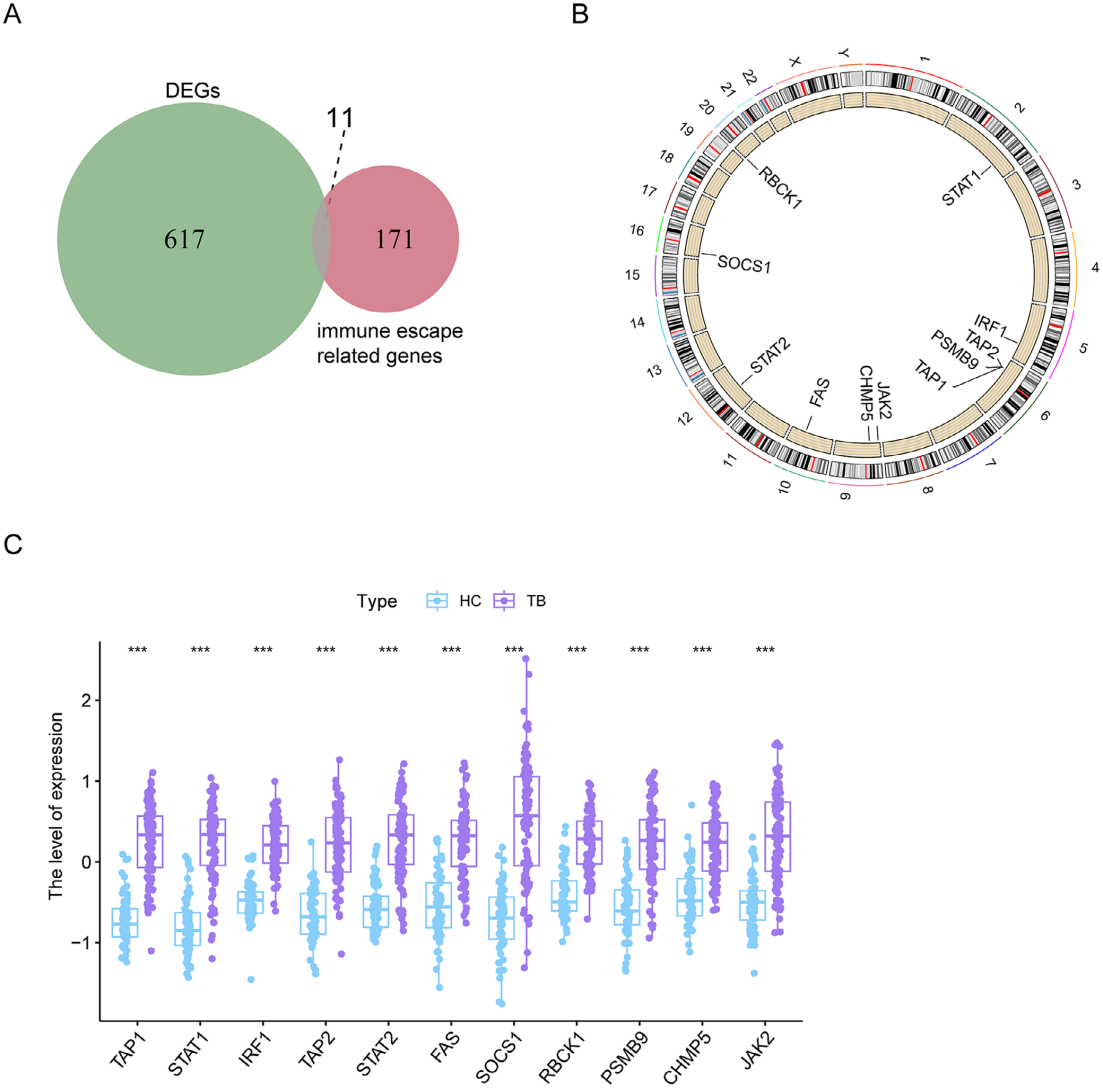
Characteristics of 11 immune escape‐related DEGs. (A) Venn plot showing the 11 immune escape‐related DEGs by intersecting the DEGs with genes. (B) The location of 11 DEGs on chromosomes. (C) Box plot showing the 11 immune escape‐related DEGs in TB samples and HC. ***p < 0.001.

Following the intersection of DEGs and the immune escape‐related genes (IERHs), we have obtained 11 IERHs, namely TAP1, STAT1, IRF1, TAP2, STAT2, FAS, SOCS1, RBCK1, PSMB9, CHMP5 and JAK2 (Figure 3A). These genes are positioned on the chromosome, as demonstrated in Figure 3B, and exhibit higher expression in TB samples compared with HC, as shown in Figure 3C.

**FIGURE 4:**
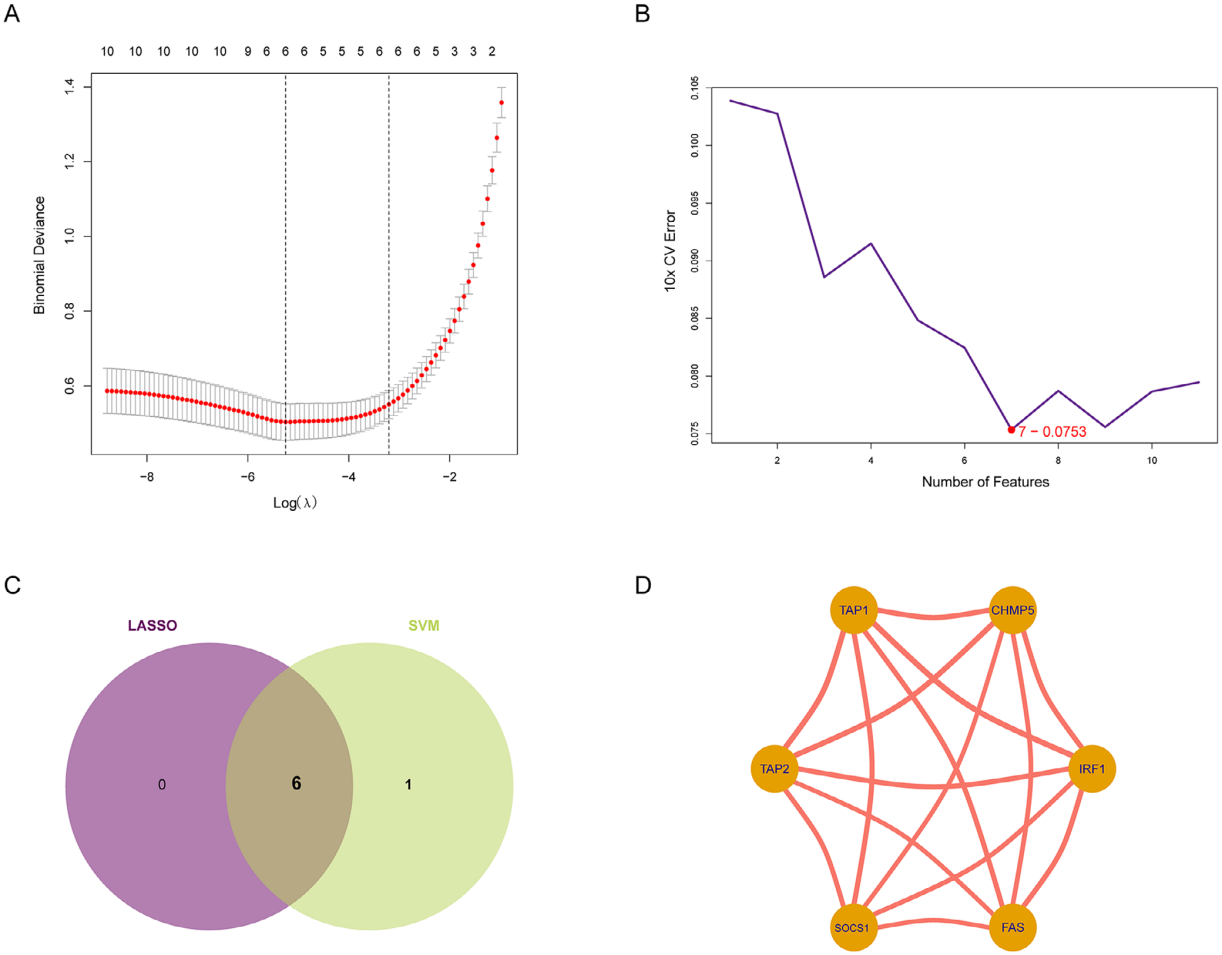
Identification of HIERGs by machine learning. (A) The optimal lambda value in the LASSO mode. (B) SVM‐RFE screening hub genes. (C) Screening of 6 HIERGs using LASSO and SVM‐RFE. (D)The relationship network of six HIERGs. HIERGs, hub immune escape‐related genes.

The LASSO regression technique and SVM were employed to screen potential genes for immune escape‐signature construction, as illustrated in Figure 4A–C, post IERHs acquisition. Finally, we have determined a six‐gene immune escape‐related signature comprising TAP1, IRF1, TAP2, FAS, SOCS1 and CHMP5 (Figure 4C). Notably, as shown in Figure 4D, the six HIERGs demonstrate a high degree of correlation with each other (cor > 0.3 and *p* < 0.05).

### 3.3 Establishment of a Nomogram Based on the Immune Escape‐Related Signature

**FIGURE 5:**
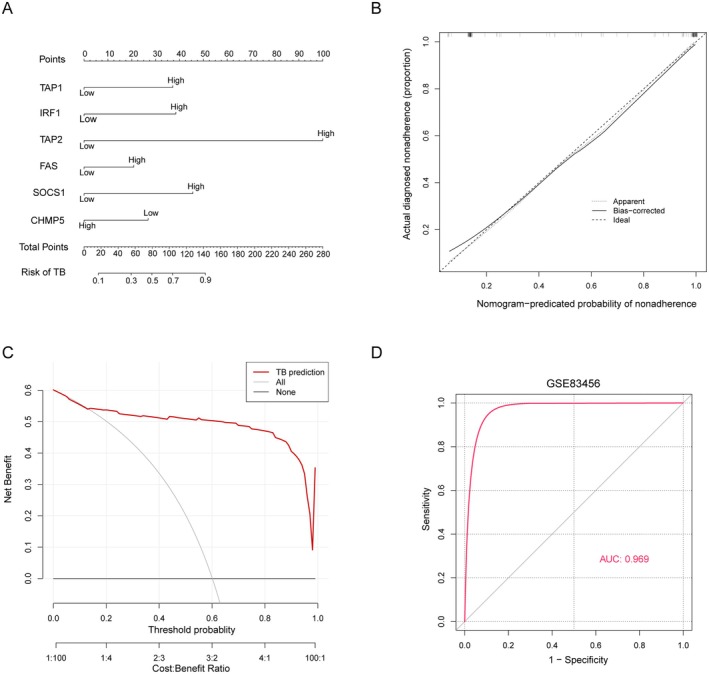
Construction of nomogram. (A) nomogram for predicting the risk of TB based on the six HIERGs. (B) Calibration plot evaluating the prediction. (C) Decision curve analysis for the nonadherence nomogram. (D) ROC curve of immune Escape‐Related Signature in TB diagnosis. AUC, srea under curve; ROC, receiver operating characteristic; Apparently, the performance or outcome of a model on the original dataset without any modification or adjustment; Bias‐corrected, corrections are made to the model to reduce or eliminate the impact of biases; Ideal, the perfect scenario where predicted values match actual values exactly.

A comprehensive nomogram, combining expression levels and clinicopathological characteristics, was constructed to provide a quantitative and easily interpretable measurement of HIERGs ability to predict the probability of TB (Figure 5A). The cohort's prediction nomogram had a C‐index of 0.9444 (95% CI: 0.90774–0.98026). The calibration curve (Figure 5B), which converged with the standard curve, a straight line with a slope of one traversing through a coordinate axis point, indicated that the nomogram signature closely corresponded to the actual survival probability. Furthermore, the decision curve (Figure 5C) demonstrated that utilising this non‐adherence nomogram to predict medication non‐adherence risk added greater benefit than the scheme with a threshold probability of > 6% for both the patient and doctor. The ROC curves of six HIERGs were examined (Figure 5D), of which the AUC was found to be 0.969, indicating that the prediction efficiency of the nomogram signature was high (n_HC_ = 61, n_TB_ = 92). Similar results were observed in the external validation cohort (GSE62525) (Figure S1).

### 3.4 WGCNA and Key Modules Analysis

**FIGURE 6:**
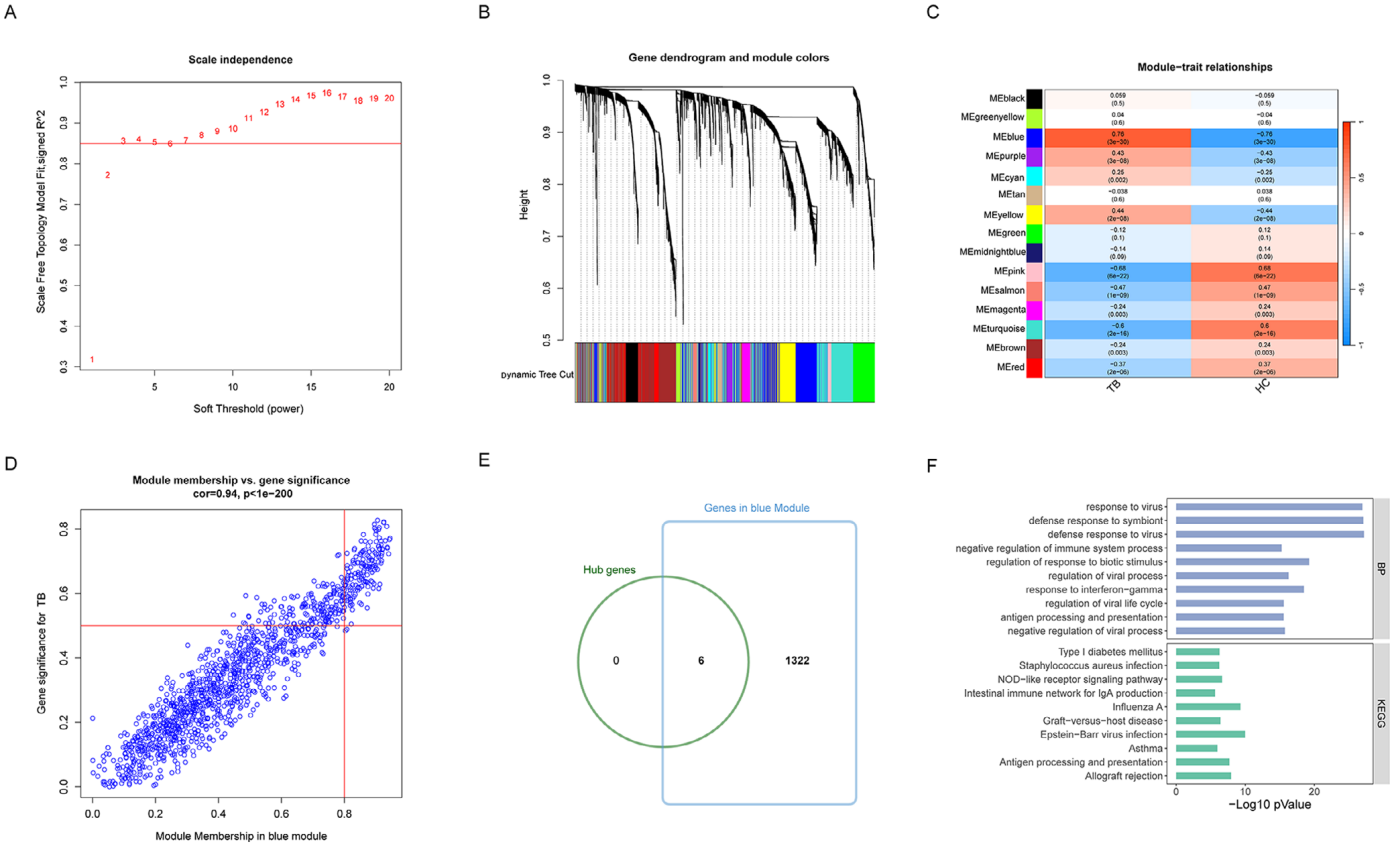
Identification of module genes in WGCNA (A) Evaluation of soft threshold power. (B) Gene dendrogram and module colours. (C) Heatmap showing the relationships between modules and features. (D) Scatter plot exhibiting the correlation between the blue module and the genes related to the module. (E) Venn plot showing the intersecting of six HIERGs and genes in blue module. (F) GO BP and KEGG analysis of the DEGs. GO BP, Gene Ontology Biological Process; KEGG, Kyoto Encyclopedia of Genes and Genomes; WGCNA, Weighted gene co‐expression network analysis.

The co‐expression network was constructed via co‐expression analysis. For this study, we included the top 20% of genes with the absolute median deviation. To ensure a scale‐free network, we selected a soft thresholding power of three, as presented in Figure 6A. Following this, we were able to identify 15 modules by means of average hierarchical clustering and dynamic tree clipping (Figure 6B,C). To highlight critical clinical modules, we examined the correlation between the network modules and external traits. As observed in Figure 6C, the blue key modules showed a significant association with TB. Additionally, the blue module was selected as the target module for subsequent analysis on the basis of its correlation coefficients r, as exhibited in Figure 6D. All HIERGs were included in the blue module (Figure 6E). These genes were then used as input for GO and KEGG analysis. Figure 6F represents the results obtained from the GO BP analysis, which showed significant enrichment of these genes in response to virus, defence response to symbiont, defence response to virus and negative regulation of immune system process. The signalling pathway enrichment showed that the genes are enriched in Type I diabetes mellitus, Intestinal immune network for IgA production, NOD–like receptor signalling pathway, *Staphylococcus aureus* infection, etc. (Figure 6F). Results of WGCNA support the above analysis.

### 3.5 The Relationship of HIERGs With Immune Cells

**FIGURE 7:**
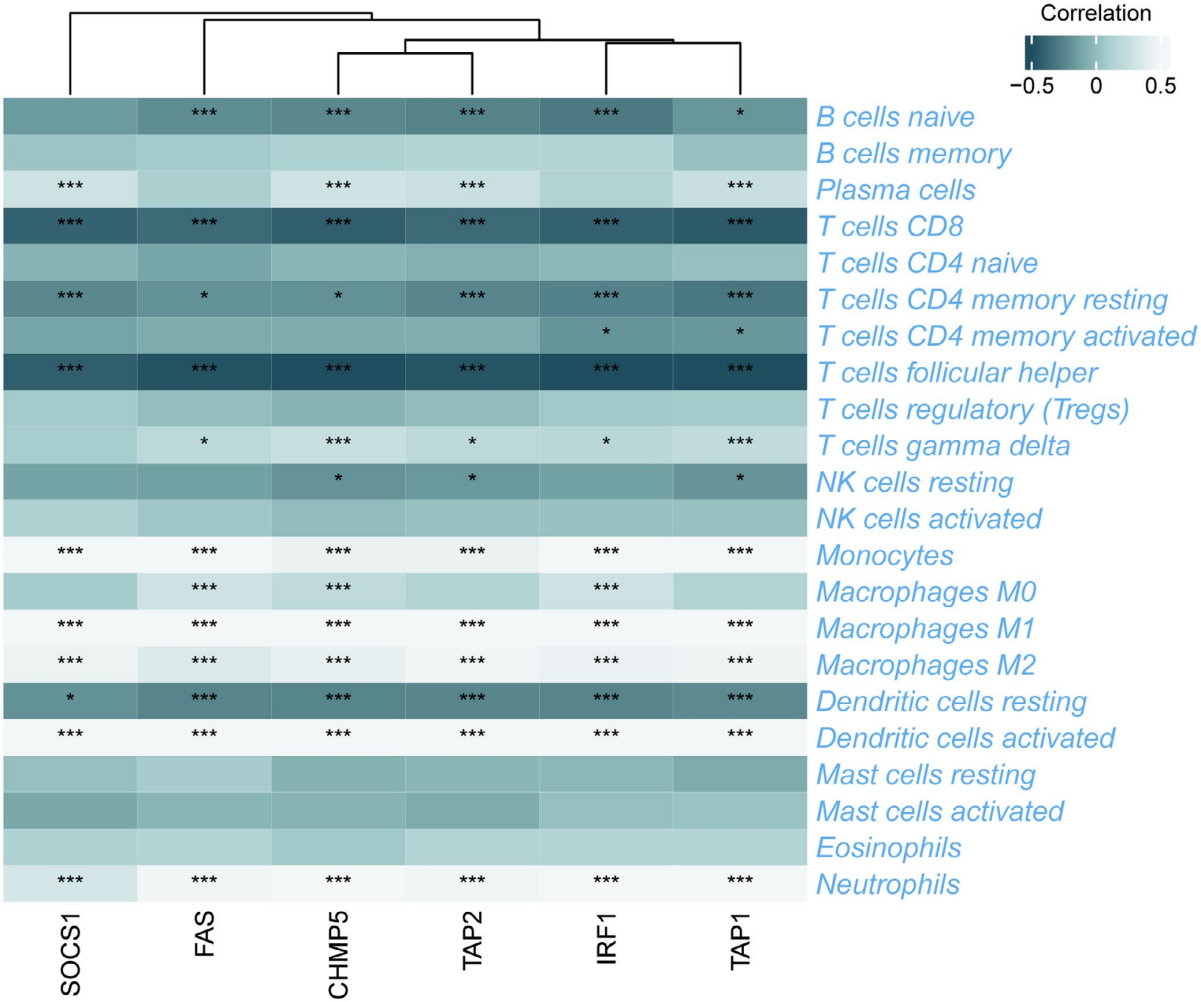
Association between six HIERGs and immune cells. *p < 0.05; ***p < 0.001.

The results above have demonstrated that immune‐related biological processes play a significant role in TB pathogenesis. To investigate the immunological regulation of TB in further detail, an immune cell infiltration analysis was conducted. As shown in Figure S2, TB samples demonstrated a higher proportion of monocytes, M2 macrophages, M1 macrophages, activated dendritic cells, eosinophils and neutrophils, while exhibiting a lower proportion of CD8+ T, resting CD4+ memory T, follicular helper T and resting NK cells. The six HIERGs displayed a significantly positive correlation with activated dendritic cells, M2 macrophages, neutrophils, M1 macrophages and monocytes, and a negative correlation with resting dendritic, CD8+ T, resting CD4+ memory T and follicular helper T cells (Figure 7).

To further investigate the relationship between HIERGs and immune cells, we utilised scRNA‐seq analysis on PBMCs derived from public datasets. These cells were classified into monocytes, neutrophils, NK, T, B and dendritic cells. The expression of TAP1, TAP2, FAS and CHMP5 genes was primarily found in monocytes, NK and T cells. IRF1 was expressed in most of the cell types analysed, while SOCS1 was found to have low expression, as illustrated in Figure S3. These results clearly reveal a strong interaction between HIERGs and immune cells.

### 3.6 Identification of Immune Escape‐Related Subtypes in TB

**FIGURE 8:**
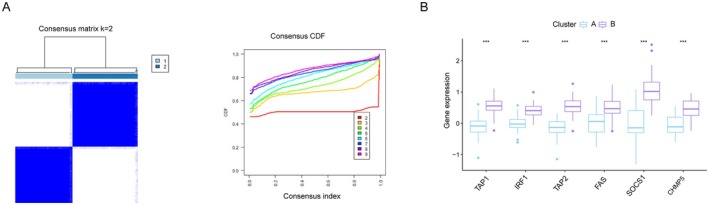
Identification of subgroups based on HIERGs. (A) Consensus clustering matrix when k = 2. (B) Boxplot of six HIERGs in the two immune escape‐related groups. ***p < 0.001.

To identify subgroups of immune escape in TB, the expression profiles of six HIERGs were analysed in 92 TB samples using a consistent unsupervised methodology. The clustering results revealed that the most stable number of subgroups was two (k = 2), as depicted in Figure 8A. The 92 TB samples were categorised into two subgroups: cluster A (*n* = 43) and cluster B (*n* = 49). Cluster B showed an enhanced expression of all six HIERGs, as shown in Figure 8B.

To further investigate the differences in pathway activity and biological functions between the two clusters, we performed GSVA analysis. Our results indicated that systemic lupus erythematosus, antigen processing and presentation, nicotinate and nicotinamide metabolism, as well as immune‐related signalling pathways, were upregulated in cluster B (Figure S4A). Additionally, Hallmark activities of interferon alpha response, interferon gamma response, allograft rejection and UV response up signalling were higher in cluster B compared with cluster A (Figure S4B). Cluster B was mainly involved in Reactome pathways, such as antigen processing cross‐presentation, regulation of IFNG signalling, pyroptosis, ovarian tumour domain proteases and interferon signalling (Figure S4C).

### 3.7 Comprehensive Analysis of DEGs and Immune Infiltration Between Immune Escape‐Related Subtypes

**FIGURE 9:**
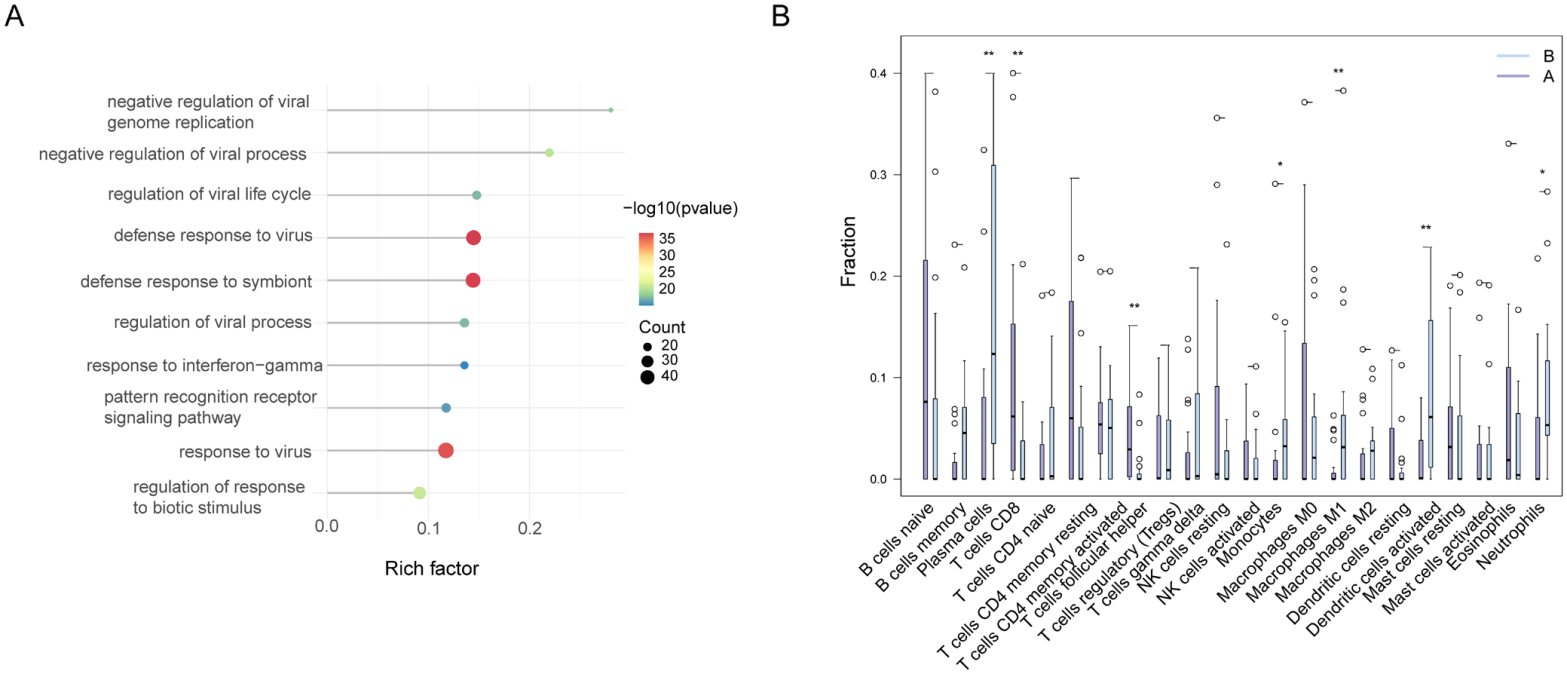
DEGs function in subgroups. (A) GO analysis of the DEGs between two clusters. (B) Comparison of immune characteristics between two clusters. *p < 0.05; **p < 0.01.

To identify the DEGs related to immune escape subgroups, we initially used the R package of limma to execute the DEGs method. From this analysis, 272 DEGs were screened, consisting of 243 upregulated DEGs and 29 downregulated DEGs, between cluster B and cluster A samples (Figure S5A). Next, GO and KEGG enrichment analyses were carried out to understand the molecular processes and functions associated with these genes. These genes were significantly enriched in the negative regulation of viral processes, negative regulation of viral genome replication, regulation of the viral life cycle, defence response to virus and response to interferon‐gamma, as indicated by GO BP analysis (Figure 9A). Furthermore, KEGG enrichment analysis demonstrated that these genes were mainly enriched in the NOD‐like receptor signalling pathway, Coronavirus disease—COVID‐19, Toll‐like receptor signalling pathway and *Staphylococcus aureus* infection (Figure S5B).

Further assessment of immune cell infiltration levels was conducted using the CIBERSORT algorithm to determine the proportions of the 22 immune cells present within each patient sample (Figure 9B). Cluster B exhibited a remarkably higher proportion of monocytes, plasma cells, M1 macrophages, activated dendritic cells and neutrophils, whereas cluster A presented more abundance of CD8+ T cells.

### 3.8 Comprehensive Analysis of Hub Genes in TB‐Related Diseases

**FIGURE 10:**
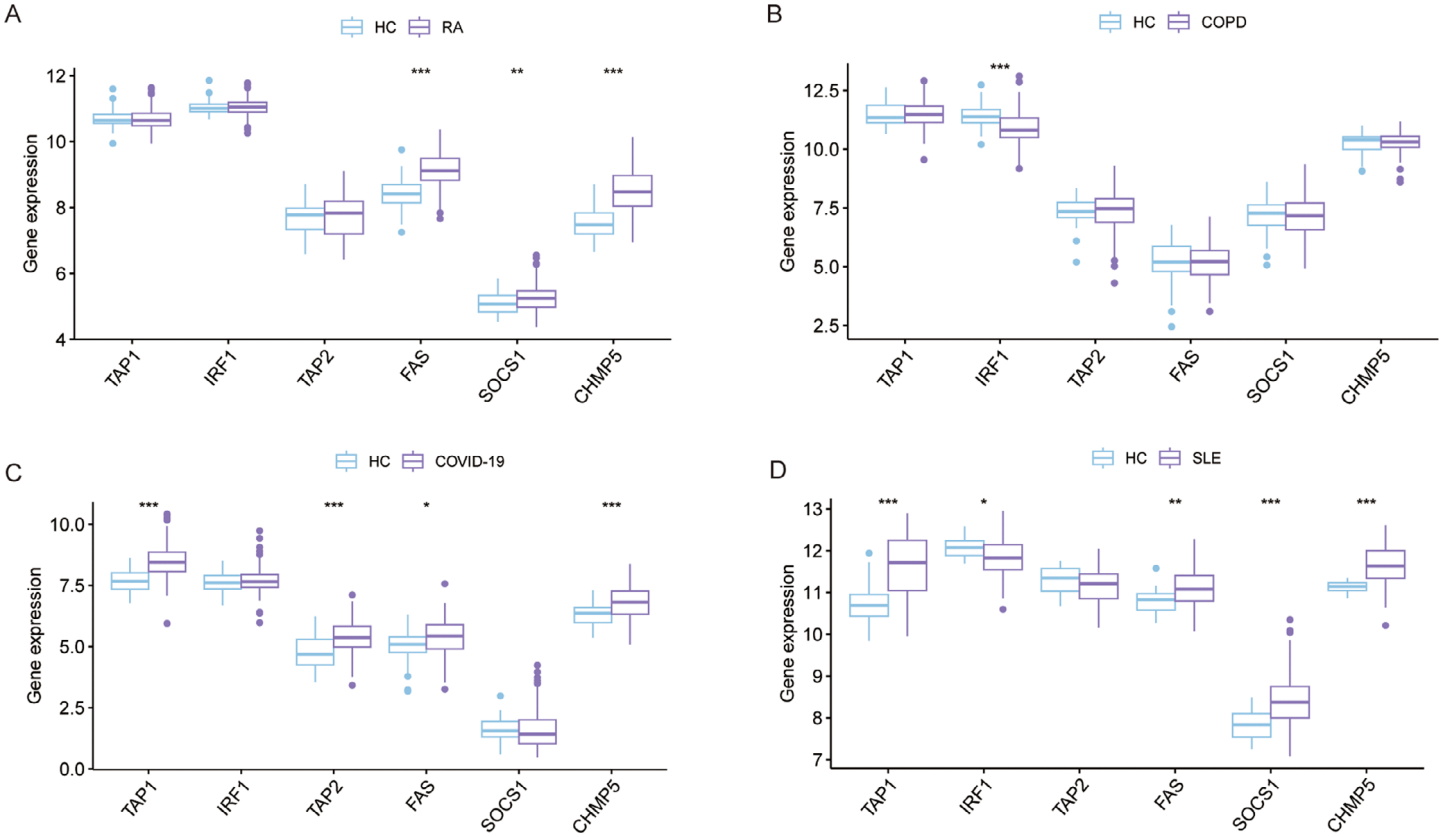
Boxplot showing the expression of six HIERGs in various diseases. *p < 0.05; **p < 0.01; ***p < 0.001. COPD, chronic obstructive pulmonary disease; RA, rheumatoid arthritis; COVID‐19, coronavirus disease; SLE, systemic lupus erythematosus.

We performed an analysis of immune infiltration in TB‐related diseases, including chronic obstructive pulmonary disease (COPD) (GSE76925) [27], rheumatoid arthritis (RA) (GSE93272) [28], coronavirus disease (COVID‐19) (GSE166253) [29], systemic lupus erythematosus (SLE) (GSE50772). As shown in Figure S6, the proportion of each type of immune cell varied within these diseases, which demonstrated the essential role played by immune cells in these diseases. Additionally, we examined the expression levels of six HIERGs in multiple diseases and results revealed that FAS and CHMP5 were significantly upregulated, except in COPD (Figure 10).

**FIGURE 11:**
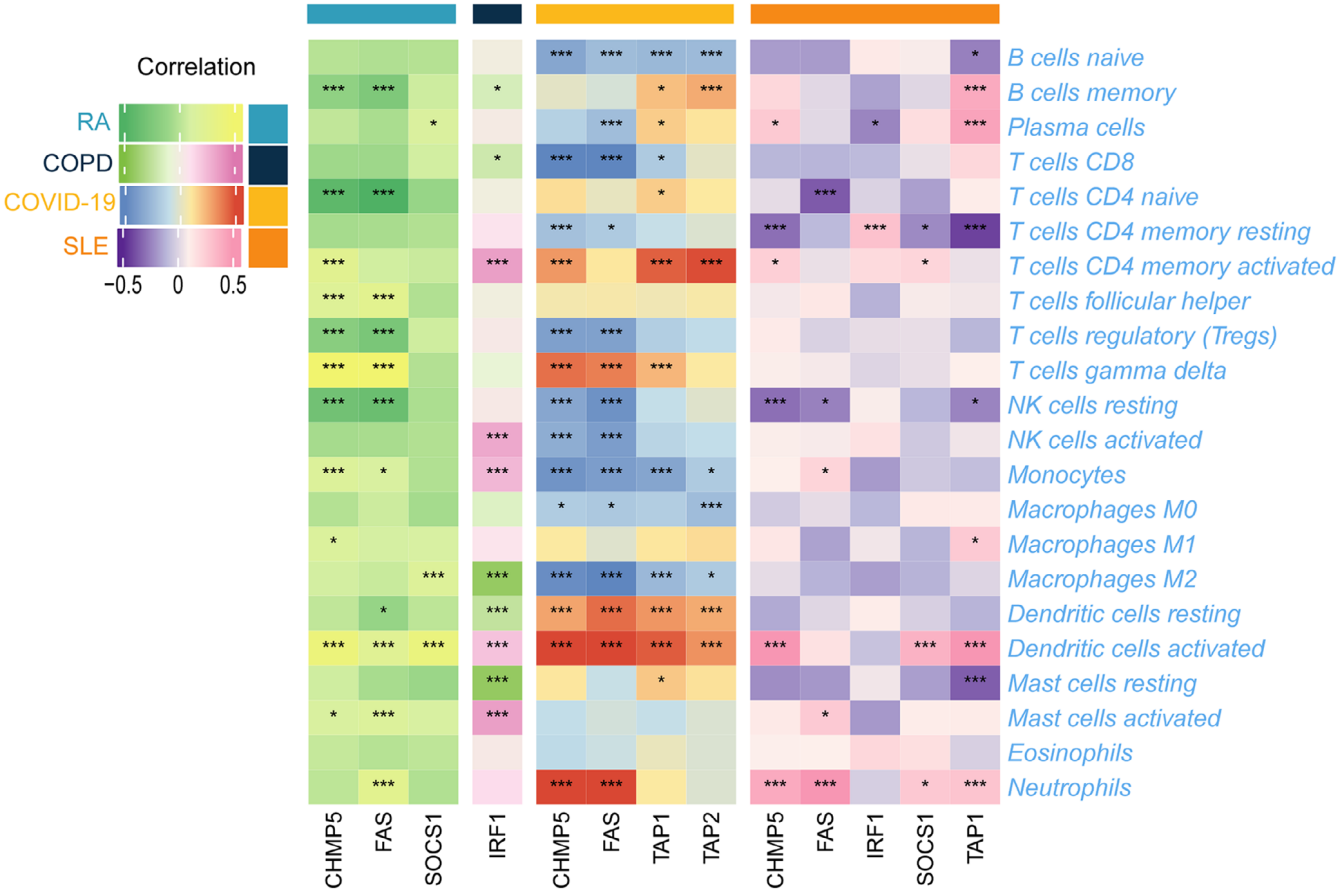
Heatmap showing association between six HIERGs and immune cells in various TB‐related diseases. * *p* < 0.05; *** *p* < 0.001. COPD, chronic obstructive pulmonary disease; RA, rheumatoid arthritis; COVID‐19, coronavirus disease; SLE, systemic lupus erythematosus.

Moreover, we examined the correlation between HIERGs and immune cells in various diseases (Figure 11). These results highlighted the significant correlation of most of these genes with neutrophils, activated dendritic cells, NK cells and other immune cells. The results revealed that the HIERGs potentially modulate the association between TB and various diseases by engaging in reciprocal interactions with immune cells.

### 3.9 Interaction of HIERGs With Chemicals

The Comparative Toxicogenomics Database (CTD, http://ctdbase.org/) was used to predict the interaction of HIERGs with chemicals [30]. Due to the high‐risk association of HIERGs with TB and its related diseases, we conducted a screening process to identify chemicals that can modulate the expression or other functionalities of these genes. In total, we identified 16, 40, 15, 141, 27 and 7 chemicals that target TAP1, IRF1, TAP2, FAS, SOCS1 and CHMP5, respectively (Table S4). These chemicals may be further utilised in clinical settings as potential drugs for treating TB and related diseases.

### 3.10 Verification of Potential Biomarker Expression by qRT‐PCR

**FIGURE 12:**
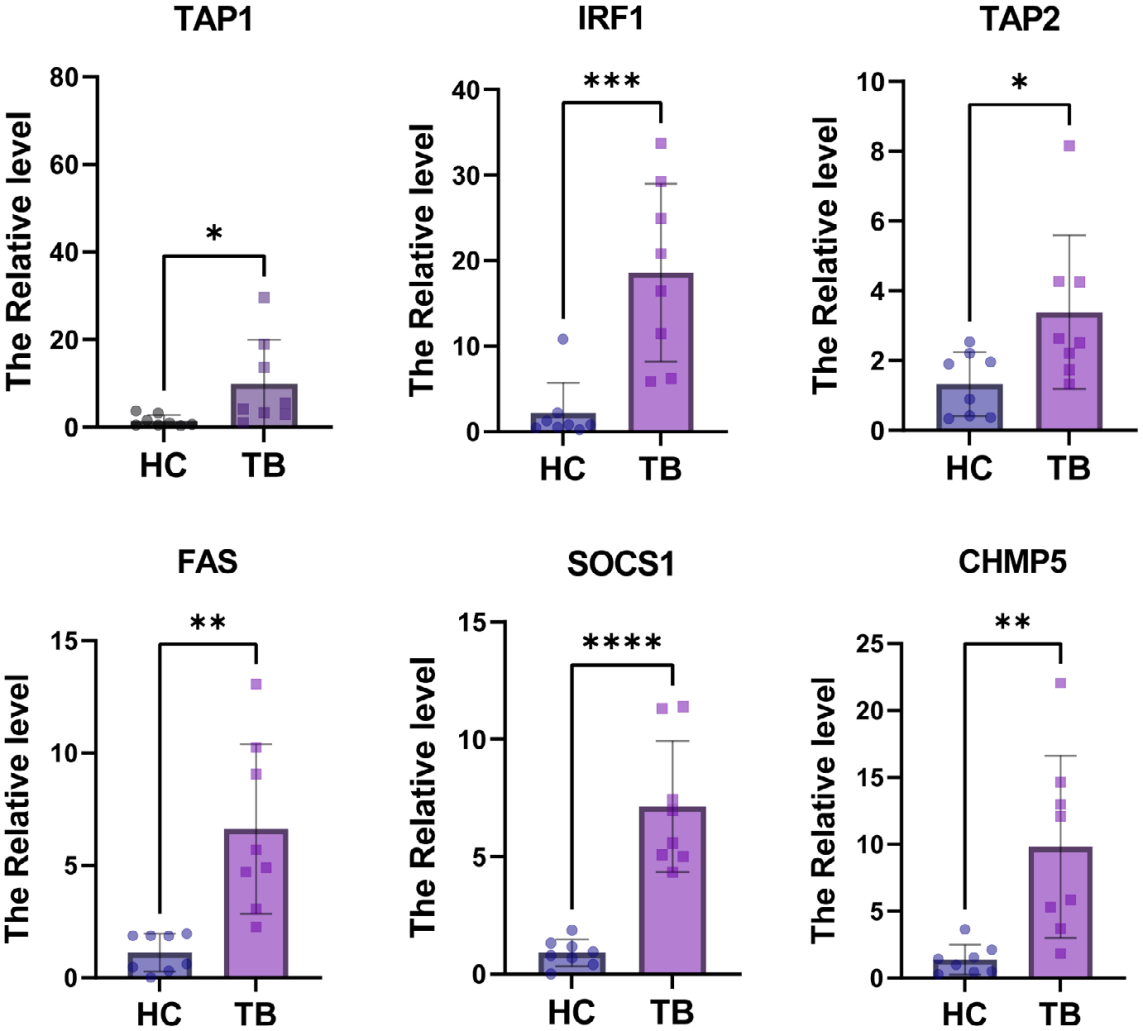
Expression level of six HIERGs in TB and HC samples. *p < 0.05; **p < 0.01; ***p < 0.001; ****p < 0.0001.

Six HIERGs were verified, and it was found that the mRNA expression level of six HIERGs was significantly higher in TB samples than in HC samples (Figure 12). The AUC of the HIERGs was 0.969 (Figure 5 D), suggesting that these hub genes could be a good a diagnostic biomarker.

## 4 Discussion

TB can be traced back to almost all of recorded human history and continues to be a leading cause of death due to infectious disease [31]. The increase in multidrug‐resistant and extensively drug‐resistant TB has led to treatment failure and increased case fatalities, which has elevated the disease's status on the priority list of the OIE and WHO [32, 33]. The severity of the threat of TB to human health has led to efforts to develop early diagnosis and treatment strategies to effectively control its global spread. In this study, we utilised bioinformatic analysis to identify immune escape‐related biomarkers, explored potential diagnostic markers for distinguishing TB from HC and examined the molecular mechanisms involved in the disease progression process. This approach improves our understanding of the heterogeneity in TB, which is essential in guiding individualised treatment of TB.

Our study identified a total of 628 DEGs in comparison with HC and TB samples. GO and KEGG analysis of the DEGs revealed that these genes were largely enriched in pathways related to viral infection and immune response. The results indicate that the host immune defence plays a critical role in controlling Mtb infection.

Through machine learning, we identified six immune escape‐associated genes (TAP1, IRF1, TAP2, FAS, SOCS1 and CHMP5) as highly effective biomarkers for TB diagnosis, demonstrating good diagnostic capability across independent datasets. TAP1 is important for MHC‐I function and has a key role in immunity to TB [34, 35, 36]. TAP2 and SOCS1 were associated with TB risk [36, 37]. IRF1 is a potential biomarker in Mtb infection [38], and FAS plays a role in immune escape in mycobacterial infection [39]. Nonetheless, additional research is required to gain a complete understanding of the significance of these genes to TB. Our WGCNA analysis produced 15 distinct clustered co‐expression modules, with the blue module showing the highest correlation coefficients and the lowest *p* value. The blue module was determined to be the most correlated module in TB progression. Further verification of these findings was achieved through hierarchical clustering of genes in the blue module, which effectively distinguished individuals in the blue group from those in the HC based on gene expression levels.

After identifying the DEGs closely related to the abnormal activation of immune functions during TB development, this study analysed the differences in immune infiltrating cells between TB and HC in blood tissues. Our findings indicated a significant correlation between the imbalance of immune cell populations, such as monocytes, M1 macrophages, M2 macrophages, activated dendritic cells, eosinophils, neutrophils, CD8+ T, resting CD4+ memory T, follicular helper T and resting NK cells with the progression of TB. Neutrophils can activate resident cells to produce inflammatory mediators, such as chemokines and cytokines, making them useful indicators of systemic inflammatory responses in various diseases [40, 41]. NK cells can directly kill Mtb‐infected cells or kill them through antibody‐dependent cellular cytotoxicity [42]. Monocytes are crucial components of innate immunity that play a critical role in the primary immune response. Studies on monocytes in TB have shown that the frequencies of intermediate and non‐classical subpopulations change, indicating their roles in persistent bacterial infections [43]. Further analysis revealed a strong correlation between HIERGs and immune cells. Single‐cell analysis confirmed these findings.

Additionally, this study classified two distinct subgroups based on the expression of six HIERGs. GSVA enrichment analysis indicated that cluster B was predominantly enriched in pathways associated with systemic lupus erythematosus, nicotinamide metabolism and immune‐related signalling pathways. Furthermore, GO analysis showed that DEGs were mainly enriched in pathways related to viral genome replication, negative regulation of viral processes, regulation of viral life cycles, defence response to viruses, and response to interferon‐gamma. KEGG analysis revealed enrichment of these genes in the NOD‐like receptor signalling pathway, Coronavirus disease‐COVID‐19 and Toll‐like receptor signalling pathway. Immune cell infiltration analysis showed that monocytes, M1 macrophages, activated dendritic cells and neutrophils had a higher proportion in cluster B, consistent with the TB and HC data mentioned in the article. Therefore, we suggest that cluster B is a high‐risk group compared with cluster A. In summary, high expression of the six HIERGs is TB risk factor.

TB is closely associated with multiple diseases, and immune cells play a crucial role in these conditions. Immune cell infiltration analysis reveals significant differences in the composition ratios of immune cells between disease samples and HC samples. Further analysis reveals that these core genes exhibit different expression patterns in different diseases and have strong interactions with immune cells. This suggests a potential association between TB and these diseases through the interaction of core genes with immune cells. Our analysis also demonstrated that these six HIERGs could serve as potential diagnostic and treatment targets for these diseases.

## 5 Conclusion

To conclude, the results of this study have demonstrated a correlation between immune escape‐related genes' expression and immune cells in TB. Moreover, this study has identified differences in immune responses between different TB patient subgroups on the basis of immune escape‐related genes. By utilising machine learning models, we have identified optimal immune escape‐related genes that can be used to precisely evaluate TB patient subtypes and guide disease diagnosis. Our findings provide novel evidence supporting the involvement of immune escape‐related genes in TB progression and offer new insights into the pathogenic basis of this disease. Furthermore, our results suggest possible approaches for improving the outcomes of infected individuals.

## Author Contributions

**Zhenpeng Li:** conceptualization (equal), data curation (equal), funding acquisition (equal), methodology (equal), validation (equal), writing – original draft (equal). **Yixin Xu:** data curation (equal), investigation (equal), methodology (equal), visualization (equal). **Huizi Zhou:** conceptualization (equal), methodology (equal), writing – original draft (equal). **Wentao Wang:** formal analysis (equal), software (equal). **Haien Cheng:** writing – review and editing (equal). **Meng Li:** visualization (equal), writing – original draft (equal). **Aili Chen:** supervision (equal), writing – review and editing (equal). **Chao Zhao:** funding acquisition (equal), supervision (equal), writing – review and editing (equal).

## Ethics Statement

This study was approved by the Ethics Committee of Weifang Medical University (2019‐036), and written informed consent was obtained from all subjects.

## Conflicts of Interest

The authors declare no conflicts of interest.

## Supporting information


**Figure S1.** (A) calibration plot evaluating the prediction in GSE62525 dataset. (B) Decision curve analysis for the nonadherence nomogram in GSE62525 dataset. (C) ROC curve of immune escape related‐signature in TB diagnosis in GSE62525 dataset.
**Figure S2.** Comparison of immune characteristics between TB and HC. *p < 0.05; **p < 0.01; ***p < 0.001.
**Figure S3.** The expression of immune escape‐related genes in different cell types.
**Figure S4.** Heatmap showing the significantly activated pathways in GSVA analysis.
**Figure S5.** DEGs function in subgroups. (A) Volcano plot of DEGs between two clusters. (B) KEGG analysis of the DEGs.
**Figure S6.** Comparison of immune characteristics between disease samples and HC in various diseases. *p < 0.05; **p < 0.01; ***p < 0.001. COPD, chronic obstructive pulmonary disease; RA, rheumatoid arthritis; COVID‐19, coronavirus disease; SLE, systemic lupus erythematosus.
**Table S1.** The GEO datasets information.
**Table S2.** Immune escape‐related genes used in this study.
**Table S3.** The markers in different cells type used in this study.
**Table S4.** Interacting chemicals of hub genes from CTD.
**Table S5.** The primers used in this study.

## Data Availability

The original contributions presented in the study are included in the article/Supporting Information. Further inquiries can be directed to the corresponding author.
