# Supplementary material for: Integrated Bioinformatic Analyses Constructed a Novel Immune Escape‐Related Signature and Classifier to Predict Tuberculosis
Source: J Cell Mol Med. 2025 Apr 27;29(8):e70562. doi: 10.1111/jcmm.70562 (PMC12034850; doi:10.1111/jcmm.70562)
Supplement: Supplementary file 1 — Figure S1. (A) calibration plot evaluating the prediction in GSE62525 dataset. (B) Decision curve analysis for the nonadherence nomogram in GSE62525 dataset. (C) ROC curve of immune escape related‐signature in TB diagnosis in GSE62525 dataset. Figure S2. Comparison of immune characteristics between TB and HC. *p < 0.05; **p < 0.01; ***p < 0.001. Figure S3. The expression of immune escape‐related genes in different cell types. Figure S4. Heatmap showing the significantly activated pathways in GSVA analysis. Figure S5. DEGs function in subgroups. (A) Volcano plot of DEGs between two clusters. (B) KEGG analysis of the DEGs. Figure S6. Comparison of immune characteristics between disease samples and HC in various diseases. *p < 0.05; **p < 0.01; ***p < 0.001. COPD, chronic obstructive pulmonary disease; RA, rheumatoid arthritis; COVID‐19, coronavirus disease; SLE, systemic lupus erythematosus. Table S1. The GEO datasets information. Table S2. Immune escape‐related genes used in this study. Table S3. The markers in different cells type used in this study. Table S4. Interacting chemicals of hub genes from CTD. Table S5. The primers used in this study. [file JCMM-29-e70562-s001.docx]

A B


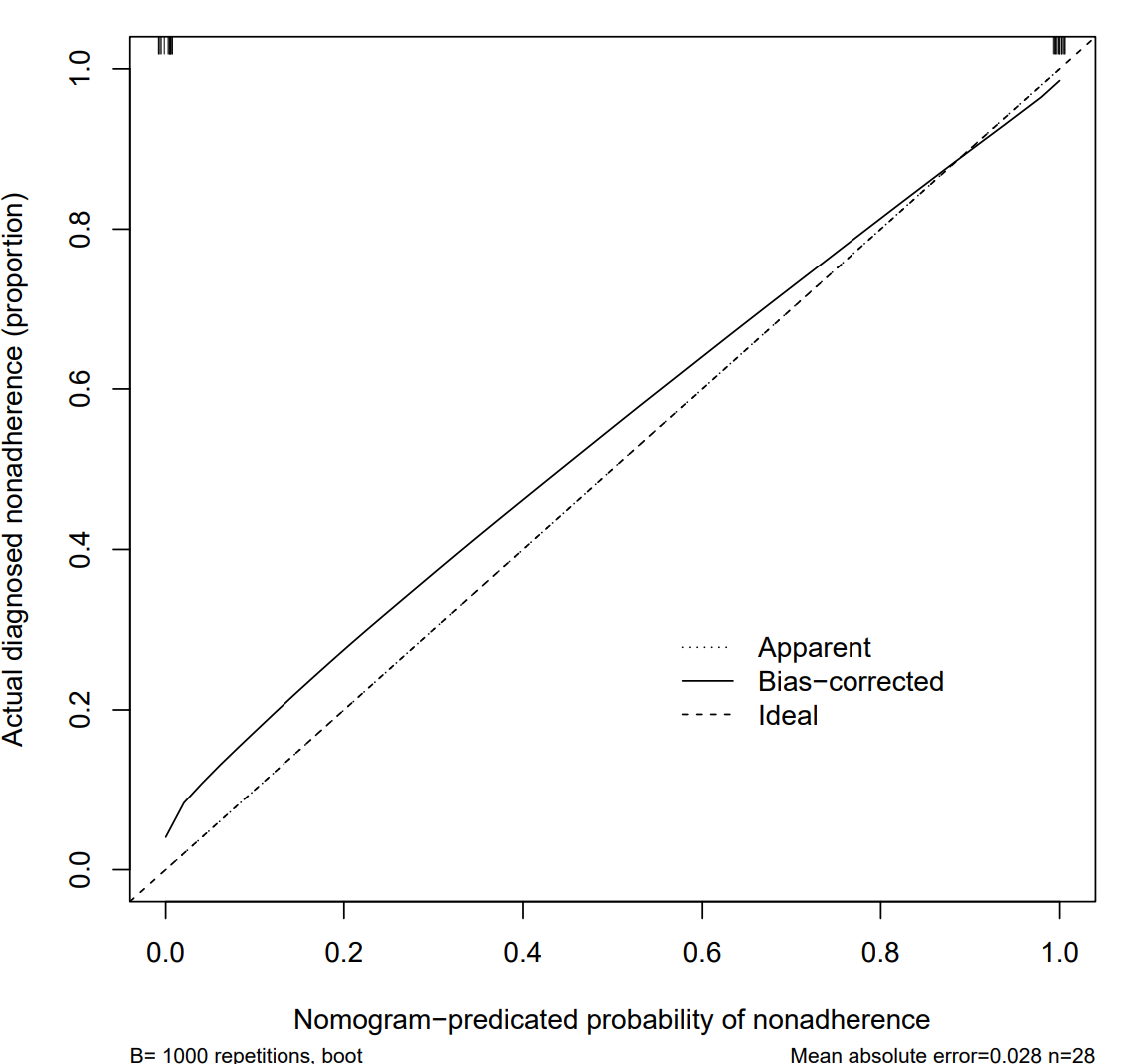

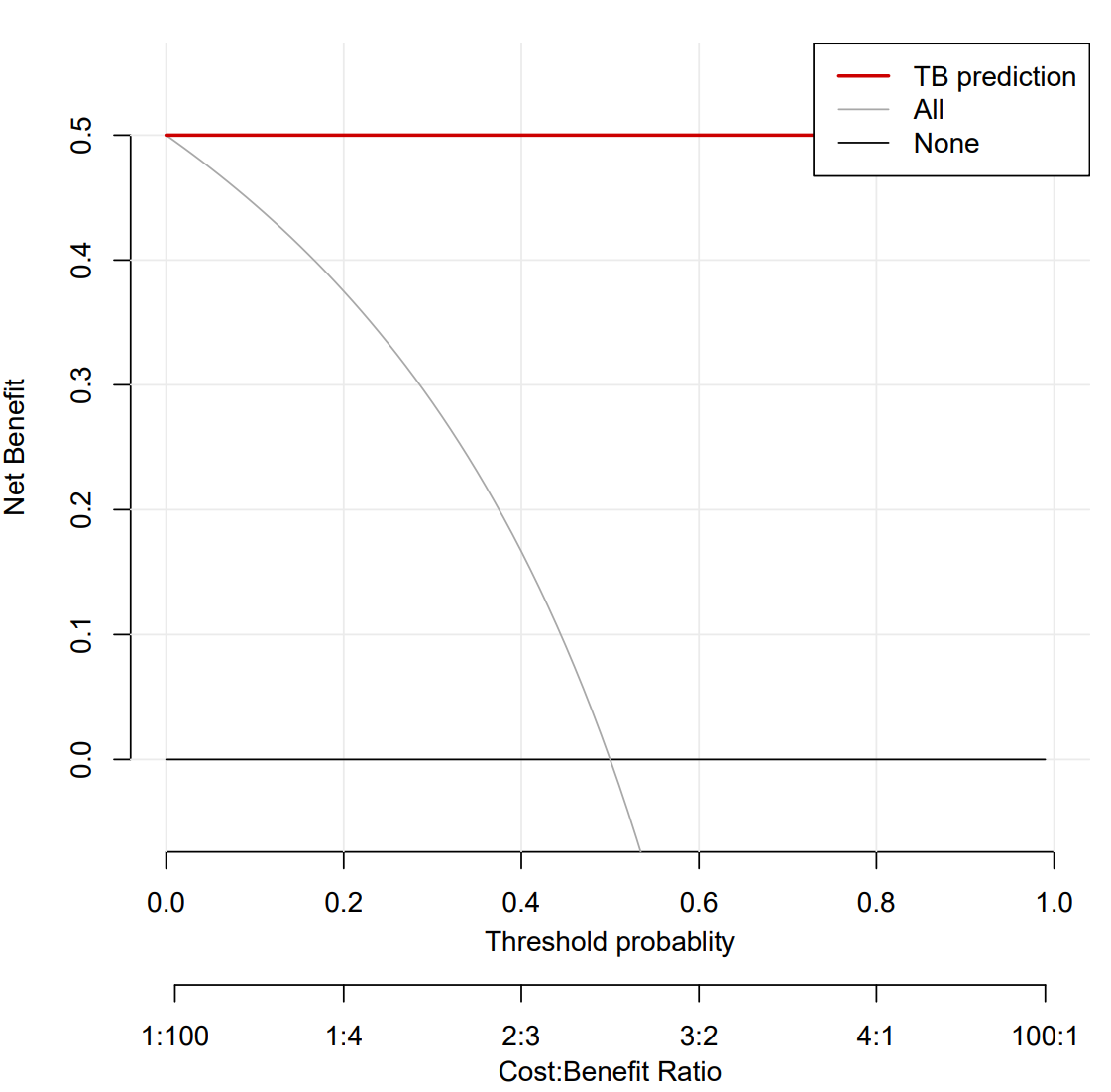


C


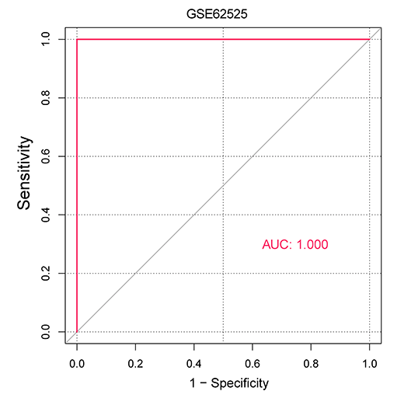


Figure S1 (A) calibration plot evaluating the prediction in GSE62525 dataset. (B) Decision curve analysis for the nonadherence nomogram in GSE62525 dataset. (C) ROC curve of immune escape related-signature in TB diagnosis in GSE62525 dataset.


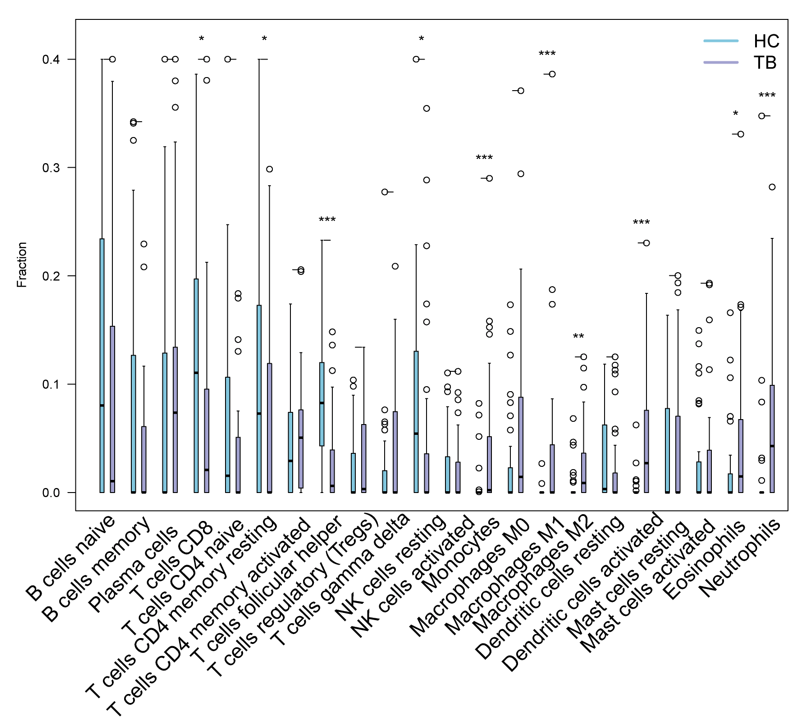


Figure S2 Comparison of immune characteristics between TB and HC. * indicated *p*<0.05; ** indicated *p*<0.01; *** indicated *p*<0.001.


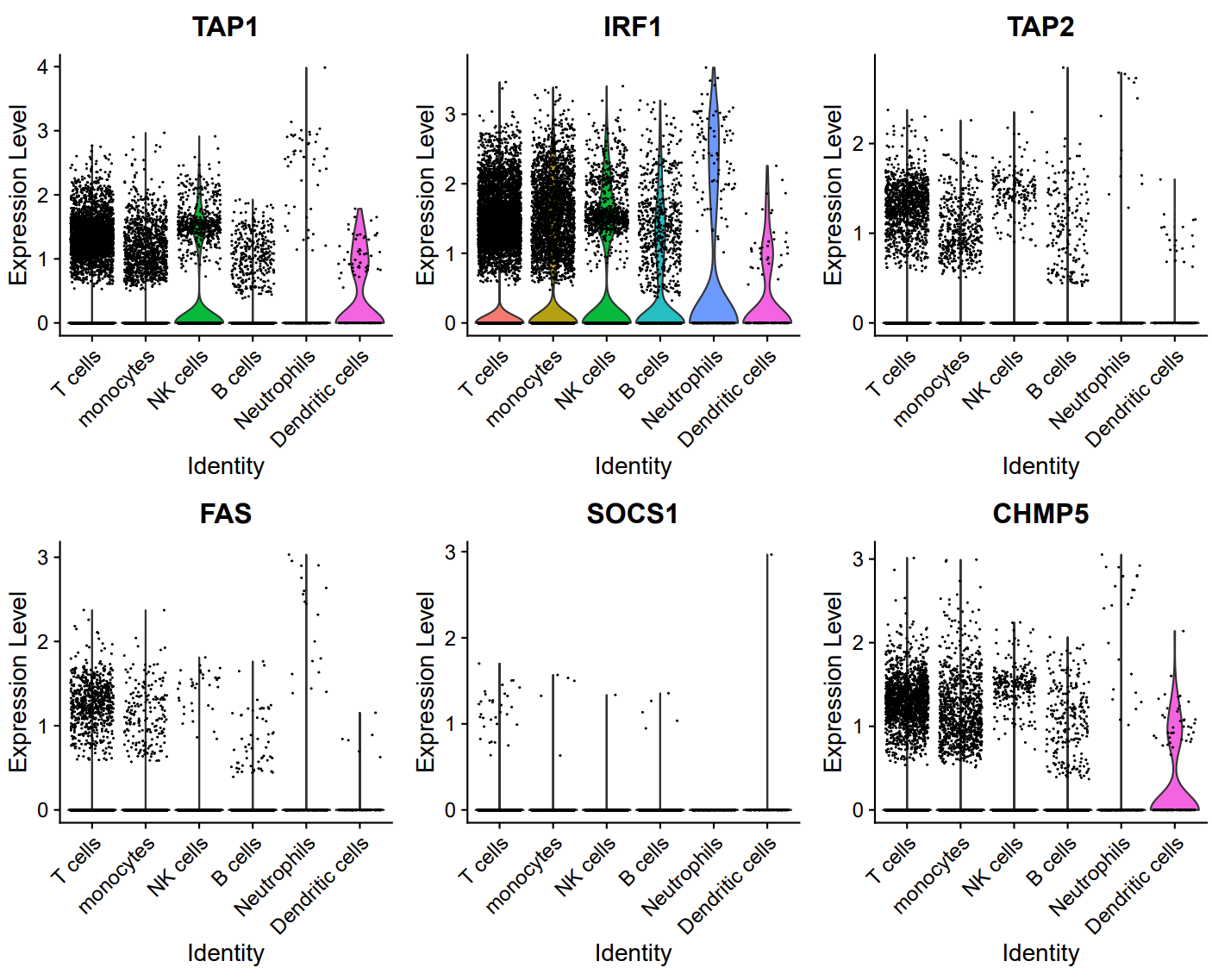


Figure S3 The expression of immune escape-related genes in different cell types.


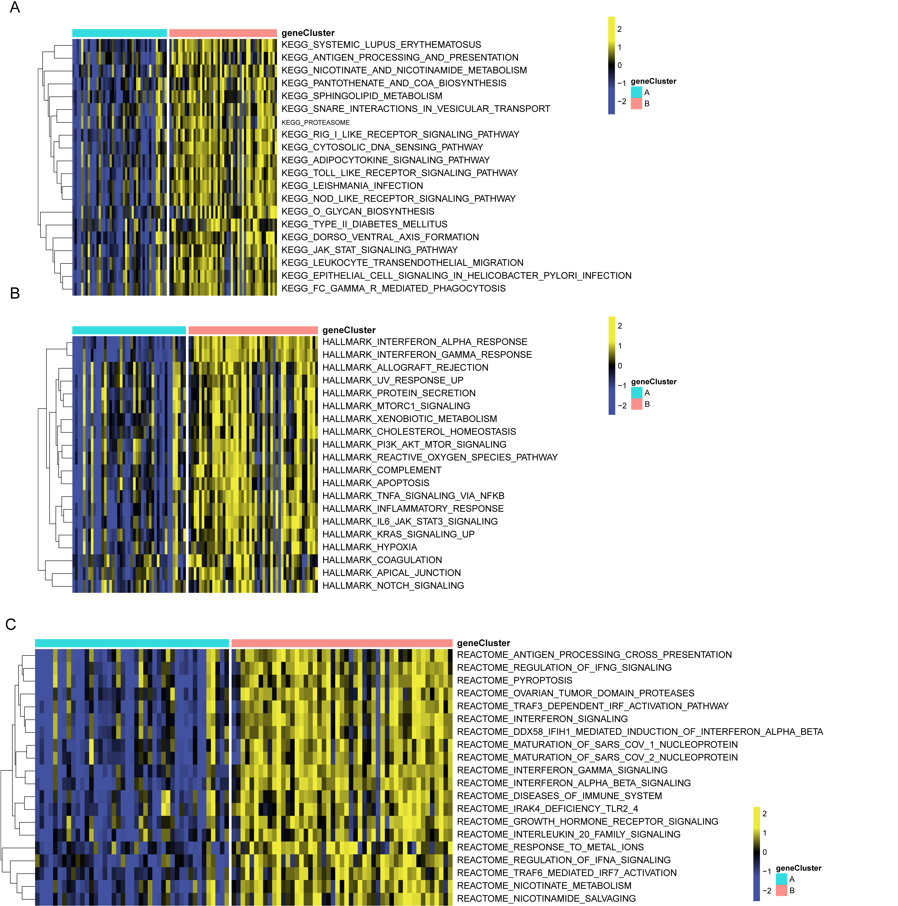


Figure S4 Heatmap showing the significantly activated pathways in GSVA analysis.

A


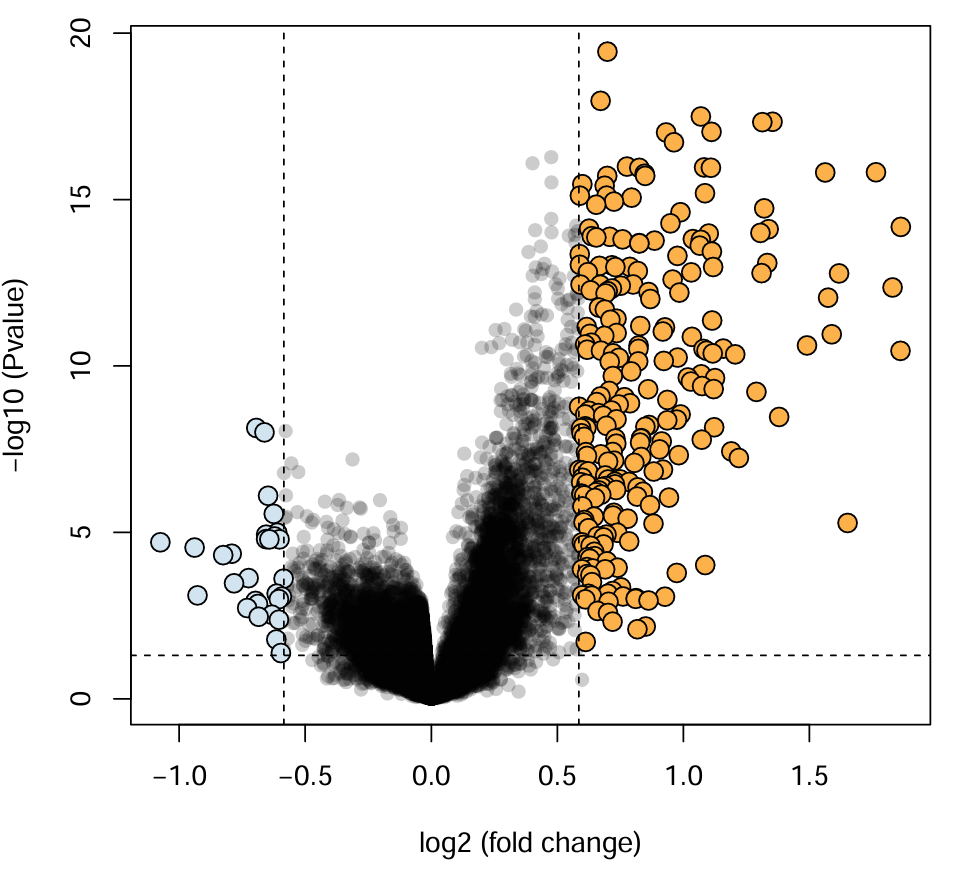


B


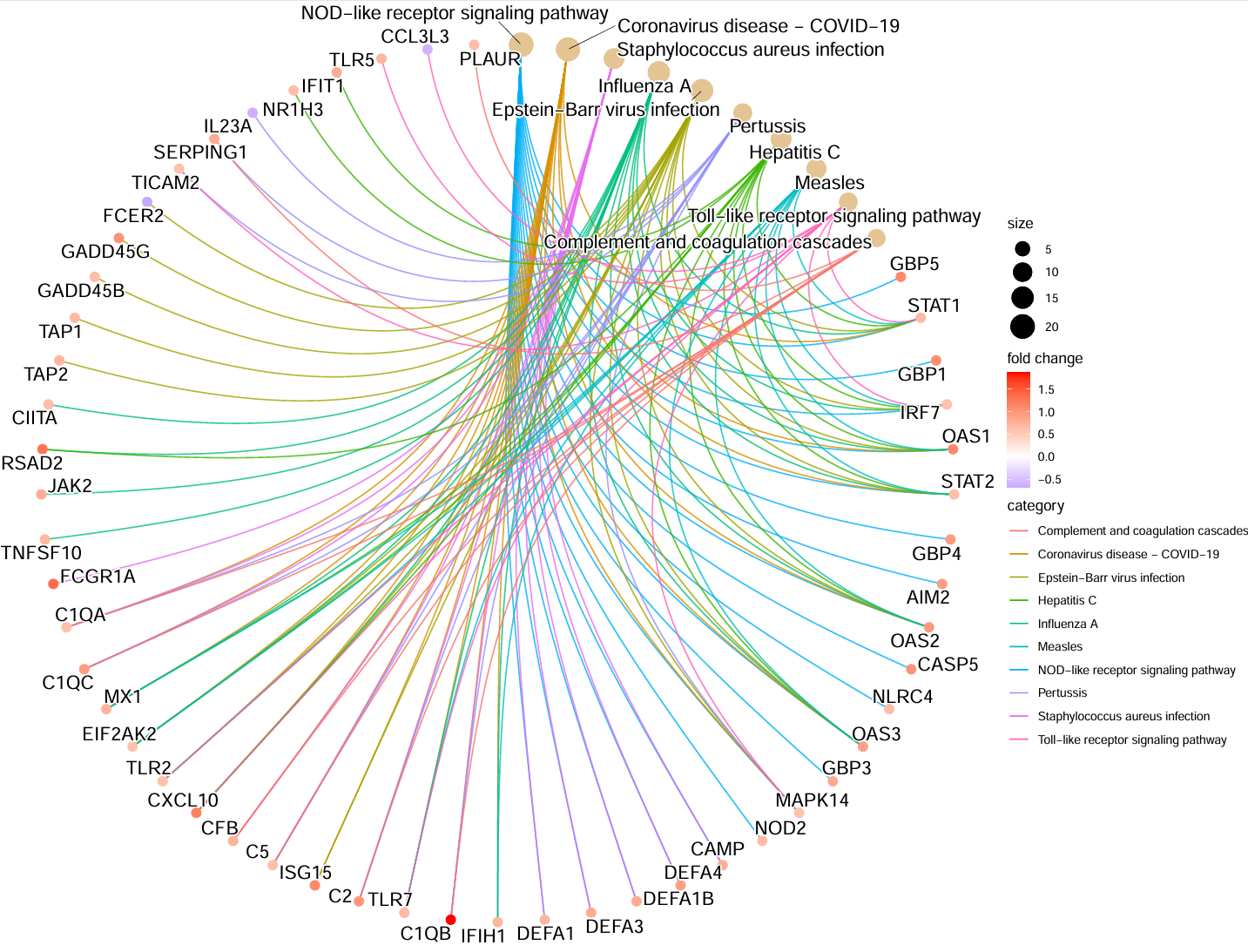


Figure S5 DEGs function in subgroups. (A) Volcano plot of DEGs between two clusters. (B) KEGG analysis of the DEGs.


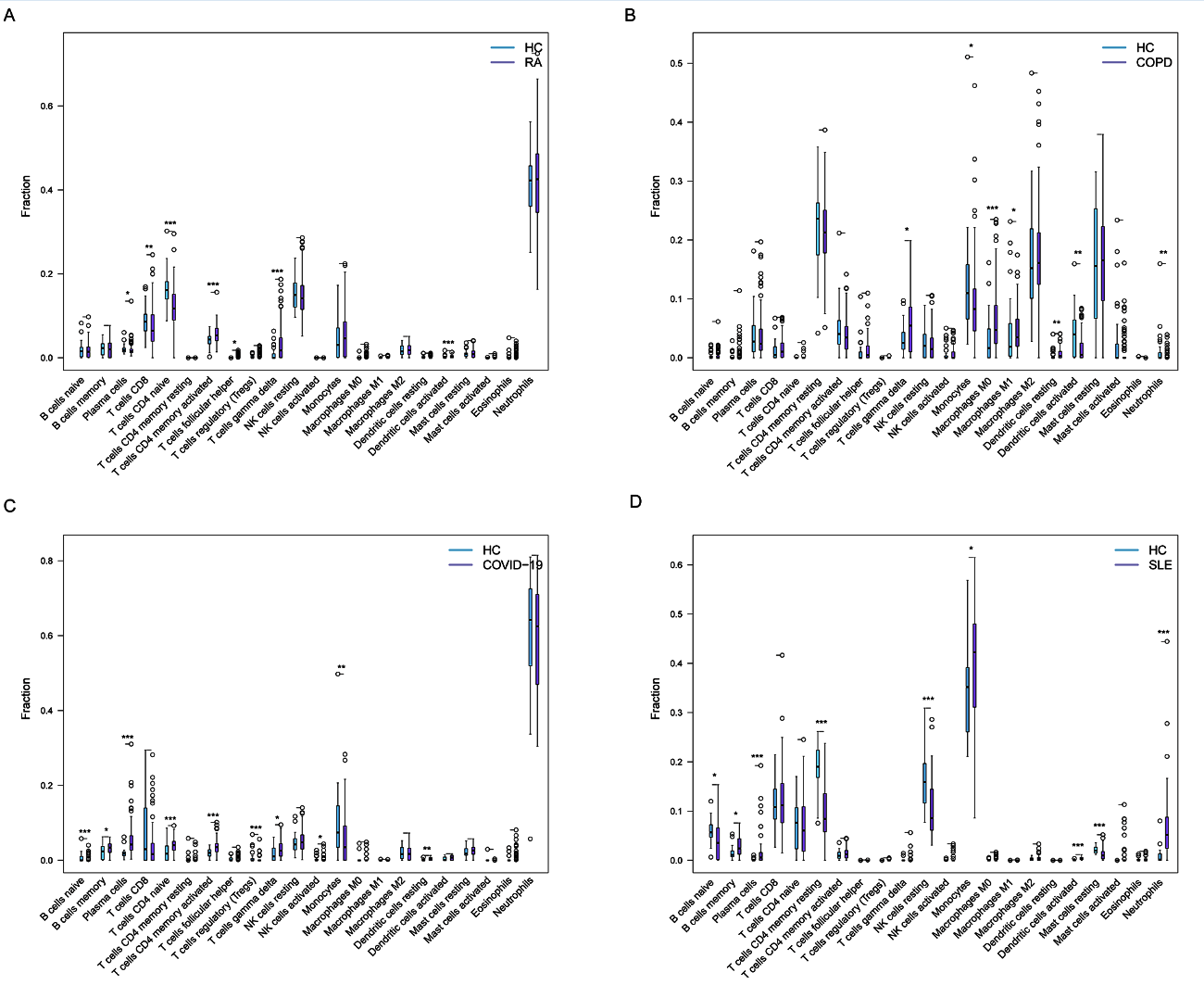


Figure S6 Comparison of immune characteristics between disease samples and HC in various diseases. * indicated *p*<0.05; ** indicated *p*<0.01; *** indicated *p*<0.001. COPD, chronic obstructive pulmonary disease; RA, rheumatoid arthritis; COVID-19, coronavirus disease; SLE, systemic lupus erythematosus.

Table S1 The GEO datasets information

| Diseases | GEO ID | Platform | HC | Case | Source | PMID |
| --- | --- | --- | --- | --- | --- | --- |
| TB | GSE83456 | GPL10558 | 61 | 92 | Blood | 27706152 |
| TB | GSE62525 | GPL16951 | 14 | 14 | PBMC | 26818387 |
| RA | GSE93272 | GPL570 | 43 | 232 | Blood | 30013029 |
| COPD | GSE76925 | GPL10558 | 40 | 111 | Lung | 28287180 |
| COVID-19 | GSE166253 | GPL20795 | 10 | 10 | PBMC | 37426635 |
| SLE | GSE50772 | GPL570 | 61 | 20 | PBMC | 25861459 |

HC, Healthy control; TB, tuberculosis; RA, rheumatoid arthritis; COVID-19, coronavirus disease; SLE, systemic lupus erythematosus.

Table S2 Immune escape-related genes used in this study

| No. | Genes |
| --- | --- |
| 1 | ACAD9 |
| 2 | ACTB |
| 3 | ADAR |
| 4 | AGO2 |
| 5 | AHSA1 |
| 6 | A/PC15 |
| 7 | ARF6 |
| 8 | ATG10 |
| 9 | ATG101 |
| 10 | ATG12 |
| 11 | ATG14 |
| 12 | ATG3 |
| 13 | ATG5 |
| 14 | ATG7 |
| 15 | ATG9A |
| 16 | ATP13A1 |
| 17 | ATXN7L3 |
| 18 | B2M |
| 19 | BC003331 |
| 20 | BCL2L1 |
| 21 | BECN1 |
| 22 | BOLA3 |
| 23 | BRAT1 |
| 24 | BRPF1 |
| 25 | CAD |
| 26 | CALR |
| 27 | CEP55 |
| 28 | CFLAR |
| 29 | CHIC2 |
| 30 | CHMP5 |
| 31 | COX6C |
| 32 | CREBBP |
| 33 | CUL3 |
| 34 | DCP1A |
| 35 | DET1 |
| 36 | DICER1 |
| 37 | D/JC13 |
| 38 | DNTTIP1 |
| 39 | DOT1L |
| 40 | DPH5 |
| 41 | EIF3H |
| 42 | EMC2 |
| 43 | EMC3 |
| 44 | EMC4 |
| 45 | EMC6 |
| 46 | EMC8 |
| 47 | ERAP1 |
| 48 | ERP44 |
| 49 | F8A |
| 50 | FADD |
| 51 | FAM58B |
| 52 | FAS |
| 53 | FITM2 |
| 54 | FNTB |
| 55 | GALE |
| 56 | GLS |
| 57 | GPAA1 |
| 58 | GPI1 |
| 59 | H2-K1 |
| 60 | HCFC2 |
| 61 | HDAC1 |
| 62 | HDGFRP2 |
| 63 | HEXIM1 |
| 64 | HIRA |
| 65 | HSPA13 |
| 66 | IF/R1 |
| 67 | IF/R2 |
| 68 | IFNGR1 |
| 69 | IFNGR2 |
| 70 | IKBKB |
| 71 | IKBKG |
| 72 | INO80 |
| 73 | IPPK |
| 74 | IRF1 |
| 75 | IRF9 |
| 76 | IST1 |
| 77 | JAGN1 |
| 78 | JAK1 |
| 79 | JAK2 |
| 80 | JMJD6 |
| 81 | KAT6A |
| 82 | KLF16 |
| 83 | KMT2A |
| 84 | LIPT2 |
| 85 | MAP3K7 |
| 86 | MAPK1 |
| 87 | MED16 |
| 88 | MED23 |
| 89 | MED24 |
| 90 | MEN1 |
| 91 | MGAT1 |
| 92 | MOGS |
| 93 | MTA2 |
| 94 | N6AMT1 |
| 95 | NCBP1 |
| 96 | NDUFA13 |
| 97 | NDUFAF6 |
| 98 | NPLOC4 |
| 99 | NUP188 |
| 100 | NXT1 |
| 101 | OTUD5 |
| 102 | OTULIN |
| 103 | PCBP2 |
| 104 | PCED1B |
| 105 | PDCD6IP |
| 106 | PDIA3 |
| 107 | PDSS2 |
| 108 | PI4KB |
| 109 | PIGK |
| 110 | PIGS |
| 111 | PIGT |
| 112 | PIGU |
| 113 | PKN2 |
| 114 | PPP1CA |
| 115 | PPP1R8 |
| 116 | PPP2R2A |
| 117 | PPP2R3C |
| 118 | PRKCSH |
| 119 | PSMB8 |
| 120 | PSMB9 |
| 121 | PSMG1 |
| 122 | PTAR1 |
| 123 | PTPN2 |
| 124 | RB1CC1 |
| 125 | RBCK1 |
| 126 | RBM15 |
| 127 | RCE1 |
| 128 | RGP1 |
| 129 | RIC1 |
| 130 | RIC8 |
| 131 | RNF31 |
| 132 | S100PBP |
| 133 | SARNP |
| 134 | SCAF4 |
| 135 | SETD1A |
| 136 | SETDB1 |
| 137 | SLC25A32 |
| 138 | SMG7 |
| 139 | SOCS1 |
| 140 | SPCS1 |
| 141 | SRRT |
| 142 | SRSF7 |
| 143 | STAT1 |
| 144 | STAT2 |
| 145 | STOML2 |
| 146 | SUSD6 |
| 147 | TAB1 |
| 148 | TAB2 |
| 149 | TAP1 |
| 150 | TAP2 |
| 151 | TAPBP |
| 152 | TBK1 |
| 153 | TFRC |
| 154 | TGFBR2 |
| 155 | TMEM127 |
| 156 | TMEM208 |
| 157 | TNFAIP3 |
| 158 | TNFRSF1A |
| 159 | TNFRSF1B |
| 160 | TRADD |
| 161 | TRAF2 |
| 162 | TRPM7 |
| 163 | UBE2G2 |
| 164 | UBE2N |
| 165 | UBR5 |
| 166 | UFC1 |
| 167 | UFL1 |
| 168 | USP7 |
| 169 | UXS1 |
| 170 | VDAC2 |
| 171 | VPS13A |
| 172 | VPS16 |
| 173 | VPS29 |
| 174 | VPS35 |
| 175 | VPS4B |
| 176 | WDR7 |
| 177 | WDR83 |
| 178 | WIPI2 |
| 179 | WWP2 |
| 180 | YAP1 |
| 181 | ZC3H3 |
| 182 | ZCCHC14 |

Table S3 The markers in different cells type used in this study

| Cells type | Markers |
| --- | --- |
| T cells | CD3D, CD3E, TRBC1 |
| NK cells | CD3D, KLRD1, NKG7, KLRC1, FCGR3A |
| B cells | MS4A1, CD79A, CD79B |
| Neutrophils | LYZ, CSF3R, CXCR2, FCGR3B |
| monocytes | LYZ, CD14, FCN1, FCGR3A, S100A9 |
| Dendritic cells | FCER1A, IL3RA, CLEC4C, LILRB4 |

| Table S4 Interacting chemicals of hub genes from CTD | |
| --- | --- |
| Genes | ChemicalName |
| tap1 | 2,2,2-trichloroethanol |
| tap1 | 7,8-Dihydro-7,8-dihydroxybenzo(a)pyrene 9,10-oxide |
| tap1 | aflatoxin G1 |
| tap1 | Antirheumatic Agents |
| tap1 | Arsenic Trioxide |
| tap1 | deoxynivalenol |
| tap1 | Dieldrin |
| tap1 | Estradiol |
| tap1 | Ivermectin |
| tap1 | perfluorohexanesulfonic acid |
| tap1 | perfluoro-n-nonanoic acid |
| tap1 | perfluorooctane sulfonic acid |
| tap1 | perfluorooctanoic acid |
| tap1 | pyrrolidine dithiocarbamic acid |
| tap1 | Smoke |
| tap1 | Tobacco Smoke Pollution |
| irf1 | 1-Methyl-3-isobutylxanthine |
| irf1 | 2-methyl-2H-pyrazole-3-carboxylic acid (2-methyl-4-o-tolylazophenyl)amide |
| irf1 | 3-((6-(2-methoxyphenyl)pyrimidin-4-yl)amino)phenyl)methane sulfonamide |
| irf1 | 4,4'-hexafluorisopropylidene diphenol |
| irf1 | Acetylcysteine |
| irf1 | Air Pollutants |
| irf1 | alpha-cyano-(3,4-dihydroxy)-N-benzylcinnamide |
| irf1 | Am 580 |
| irf1 | Antirheumatic Agents |
| irf1 | Arsenic |
| irf1 | Arsenicals |
| irf1 | Arsenic Trioxide |
| irf1 | Benzo(a)pyrene |
| irf1 | beta-hydroxy simvastatin acid |
| irf1 | beta-Naphthoflavone |
| irf1 | bisphenol A |
| irf1 | Calcitriol |
| irf1 | caryophyllene |
| irf1 | Dactinomycin |
| irf1 | Dexamethasone |
| irf1 | Dimethyl Fumarate |
| irf1 | Dronabinol |
| irf1 | Estradiol |
| irf1 | Lipopolysaccharides |
| irf1 | Menthol |
| irf1 | monomethylarsonous acid |
| irf1 | nickel sulfate |
| irf1 | N,N,N',N'-tetrakis(2-pyridylmethyl)ethylenediamine |
| irf1 | Puromycin |
| irf1 | pyrrolidine dithiocarbamic acid |
| irf1 | Ro 41-5253 |
| irf1 | Rosiglitazone |
| irf1 | ruxolitinib |
| irf1 | Silver |
| irf1 | SR 144528 |
| irf1 | Sunitinib |
| irf1 | Tetrachlorodibenzodioxin |
| irf1 | Thioctic Acid |
| irf1 | Tosyllysine Chloromethyl Ketone |
| irf1 | Tretinoin |
| tap2 | abrine |
| tap2 | Air Pollutants, Occupational |
| tap2 | Antirheumatic Agents |
| tap2 | bisphenol A |
| tap2 | Cadmium |
| tap2 | Copper |
| tap2 | Disulfiram |
| tap2 | Doxorubicin |
| tap2 | Ivermectin |
| tap2 | Methyl Methanesulfonate |
| tap2 | monomethylarsonous acid |
| tap2 | Nickel |
| tap2 | Oxygen |
| tap2 | Smoke |
| tap2 | Thimerosal |
| fas | 10-methoxy-2,2-dimethyl-2,6-dihydropyrano(3,2-c)quinolin-5-one |
| fas | 1,2-bis(2-aminophenoxy)ethane N,N,N',N'-tetraacetic acid acetoxymethyl ester |
| fas | 15-deoxy-delta(12,14)-prostaglandin J2 |
| fas | 2-(2-amino-3-methoxyphenyl)-4H-1-benzopyran-4-one |
| fas | 2,3,5-trichloro-6-phenyl-(1,4)benzoquinone |
| fas | 2'-(4-chlorophenyl)-3-(4-chlorophenylidene)-5,10-dimethyl-12-methylene-decahydro-1,2-(1',3'-dioxocyclopenta(c))azuleno(4,5-b)furan-4,11-dione |
| fas | 2-(4-morpholinyl)-8-phenyl-4H-1-benzopyran-4-one |
| fas | 2-bromopalmitate |
| fas | 2-morpholin-4-yl-6-thianthren-1-yl-pyran-4-one |
| fas | 3-(3-chloro-4-hydroxyphenylamino)-4-(4-nitrophenyl)-1H-pyrrole-2,5-dione |
| fas | 4-(4-fluorophenyl)-2-(4-hydroxyphenyl)-5-(4-pyridyl)imidazole |
| fas | 4-hydroxyisoleucine |
| fas | 4-oxoretinoic acid |
| fas | 4-oxoretinol |
| fas | 5-OH-BDE-47 |
| fas | 7,8-Dihydro-7,8-dihydroxybenzo(a)pyrene 9,10-oxide |
| fas | Acetaminophen |
| fas | Acetylcysteine |
| fas | Acrolein |
| fas | Aflatoxin B1 |
| fas | Air Pollutants |
| fas | Alitretinoin |
| fas | Antimycin A |
| fas | apicidin |
| fas | Arachidonic Acid |
| fas | Arsenic Trioxide |
| fas | Asbestos |
| fas | asparanin A |
| fas | Aspirin |
| fas | Atenolol |
| fas | Benzo(a)pyrene |
| fas | benzyloxycarbonyl-valyl-alanyl-aspartic acid |
| fas | Biological Products |
| fas | bisphenol A |
| fas | Bortezomib |
| fas | butylphen |
| fas | Butyric Acid |
| fas | cacalol |
| fas | Cacodylic Acid |
| fas | cadmium acetate |
| fas | Cadmium Chloride |
| fas | Calcimycin |
| fas | Camptothecin |
| fas | Cannabidiol |
| fas | Carvedilol |
| fas | Cholesterol |
| fas | Cisplatin |
| fas | Clodronic Acid |
| fas | Copper Sulfate |
| fas | corosolic acid |
| fas | Cyclophosphamide |
| fas | Cyclosporine |
| fas | Dexamethasone |
| fas | Diazinon |
| fas | Diosgenin |
| fas | Docosahexaenoic Acids |
| fas | dorsomorphin |
| fas | Doxorubicin |
| fas | Epinephrine |
| fas | Estradiol |
| fas | Etoposide |
| fas | Fenofibrate |
| fas | Fluorouracil |
| fas | Fructose |
| fas | fulvic acid |
| fas | Genistein |
| fas | ginsenoside Re |
| fas | Glucose |
| fas | Gold |
| fas | Heterocyclic Compounds |
| fas | Hydrogen Peroxide |
| fas | Ibuprofen |
| fas | isosilybin |
| fas | Ivermectin |
| fas | jinfukang |
| fas | (+)-JQ1 compound |
| fas | Ketoconazole |
| fas | Leukotriene D4 |
| fas | Lipopolysaccharides |
| fas | Lithium |
| fas | Lovastatin |
| fas | Magnesium Oxide |
| fas | Melatonin |
| fas | Menthol |
| fas | Mercuric Chloride |
| fas | Mercury |
| fas | Methotrexate |
| fas | Mifepristone |
| fas | monomethylarsonic acid |
| fas | monomethylarsonous acid |
| fas | moringin |
| fas | Nanotubes, Carbon |
| fas | Nitrogen Oxides |
| fas | nodularin |
| fas | Nystatin |
| fas | ochratoxin A |
| fas | Oleic Acid |
| fas | Orlistat |
| fas | Paclitaxel |
| fas | Palmitic Acid |
| fas | parthenolide |
| fas | Particulate Matter |
| fas | Pemetrexed |
| fas | Pentoxifylline |
| fas | perfluorodecanoic acid |
| fas | perfluorohexanesulfonic acid |
| fas | perfluoroundecanoic acid |
| fas | Permethrin |
| fas | pifithrin |
| fas | platycodin D |
| fas | Polyethyleneimine |
| fas | pyrazolanthrone |
| fas | Pyrethrins |
| fas | raltitrexed |
| fas | Resveratrol |
| fas | Rifampin |
| fas | Rotenone |
| fas | S-(1,2-dichlorovinyl)cysteine |
| fas | S-allylcysteine |
| fas | SB 203580 |
| fas | Selenium |
| fas | Smoke |
| fas | sodium arsenite |
| fas | Sorafenib |
| fas | STO 609 |
| fas | Tacrolimus |
| fas | Tetradecanoylphorbol Acetate |
| fas | Thalidomide |
| fas | Thiazoles |
| fas | Thioctic Acid |
| fas | Tobacco Smoke Pollution |
| fas | Topotecan |
| fas | Tretinoin |
| fas | Tubocurarine |
| fas | Urethane |
| fas | Valproic Acid |
| fas | Vitamin A |
| fas | Vorinostat |
| fas | Y 27632 |
| fas | Zinc Oxide |
| fas | ziyuglycoside II |
| socs1 | 4-oxoretinoic acid |
| socs1 | 7,8-Dihydro-7,8-dihydroxybenzo(a)pyrene 9,10-oxide |
| socs1 | Acetaminophen |
| socs1 | afimoxifene |
| socs1 | Alitretinoin |
| socs1 | Arsenic Trioxide |
| socs1 | beta-hydroxy simvastatin acid |
| socs1 | Bleomycin |
| socs1 | Carmustine |
| socs1 | Cisplatin |
| socs1 | Copper Sulfate |
| socs1 | Cyclosporine |
| socs1 | Decitabine |
| socs1 | Estradiol |
| socs1 | Fulvestrant |
| socs1 | gardiquimod |
| socs1 | GSK-J4 |
| socs1 | Isotretinoin |
| socs1 | Lomustine |
| socs1 | Progesterone |
| socs1 | Protein Kinase Inhibitors |
| socs1 | Resveratrol |
| socs1 | sodium arsenite |
| socs1 | Tetrachlorodibenzodioxin |
| socs1 | Tretinoin |
| socs1 | tris(1,3-dichloro-2-propyl)phosphate |
| socs1 | vanadium pentoxide |
| chmp5 | arsenic disulfide |
| chmp5 | arsenic trisulfide |
| chmp5 | Caffeine |
| chmp5 | dicrotophos |
| chmp5 | Enzyme Inhibitors |
| chmp5 | Estradiol |
| chmp5 | Ivermectin |

Table S5 The primers used in this study

|  | Forward Primer | Reverse Primer |
| --- | --- | --- |
| IRF1 | CTGTGCGAGTGTACCGGATG | ATCCCCACATGACTTCCTCTT |
| TAP1 | TGCCCCGCATATTCTCCCT | CACCTGCGTTTTCGCTCTTG |
| TAP2 | TGGACGCGGCTTTACTGTG | GCAGCCCTCTTAGCTTTAGCA |
| FAS | TCTGGTTCTTACGTCTGTTGC | CTGTGCAGTCCCTAGCTTTCC |
| SOCS1 | TTTTCGCCCTTAGCGTGAAGA | GAGGCAGTCGAAGCTCTCG |
| CHMP5 | AGATTTCTCGATTGGATGCTGAG | TGTTGGGCAAGATTGTCCCG |
| GAPDH | ACAACTTTGGTATCGTGGAAGG | GCCATCACGCCACAGTTTC |
